# Semantic congruence impacts audiovisual processing in the Colavita effect

**DOI:** 10.1371/journal.pone.0352876

**Published:** 2026-07-06

**Authors:** Keira Dyck, Abigail Mitchell, Jonathan Wilbiks, Geneviève Desmarais

**Affiliations:** 1 Department of Psychology, Mount Allison University, Sackville, New Brunswick, Canada; 2 Department of Psychology, University of New Brunswick Saint John, Saint John, New Brunswick, Canada; University of Bologna, ITALY

## Abstract

The Colavita effect is a phenomenon that occurs when participants are presented with auditory, visual, and audiovisual stimuli and are tasked to identify the modality of the stimuli. When audiovisual stimuli are presented, participants occasionally miss the auditory component and report that the stimulus was visual only – reflecting visual dominance. The Colavita effect has been shown to be resistant to manipulations of semantic correspondence, which is surprising considering that semantic information impacts multiple perceptual tasks. It is possible that the brief auditory stimuli used for Colavita tasks do not allow for recognition, preventing the recruitment of semantics. We first replicated the Colavita effect using words as stimuli. We then manipulated semantic correspondence by pairing the auditory (sounds or words) and visual (pictures) components of the stimuli either with the same concept (e.g., the picture of a cat with the sound of a cat or the word ‘cat’) or different concepts (e.g., the picture of a cat with the sound of a dog or the word ‘dog’). In Experiment 2, we observed that semantic correspondence influenced the magnitude of the Colavita effect: when the visual and auditory component corresponded to different concepts, the size of the Colavita effect was reduced. Finally, in Experiment 3 we observed that using visual and auditory stimuli from different categories further reduced the effect. We suggest semantic congruence influences the unity of bimodal stimuli: congruent stimuli are more easily perceived as a single entity while incongruent stimuli are not as easily perceived as a single entity, leading to a reduction of ‘visual-only’ responses.

## 1. Introduction

The Colavita effect is a phenomenon where individuals asked to report the modality of stimuli fail to detect the auditory component of audiovisual stimuli and report that audiovisual stimuli were ‘visual only’ more often than they report that they were ‘auditory only’ [1,2]. The Colavita effect is a reliably demonstrated effect [3–5], persisting through manipulations of response characteristics including: number of keys used [2], number of targets requiring a response [6], proportion of trials in different modalities [2,6,7], stimulus intensity [1], the addition of a secondary task [8], and the type of stimulus being presented (e.g., audio-tactile, visuo-tactile) [9,10].

Importantly, the Colavita effect has been observed with different kinds of stimuli: abstract stimuli like flashes paired with tones [1,7], line drawings paired with animal noises [6], and coloured photographs paired with animal noises [8,11]. Koppen et al. [11] reported that the Colavita effect observed using concrete stimuli like pictures of animals is typically stronger than the effect observed when abstract stimuli (e.g., * or Ͼ) are presented, and suggested that the concrete stimuli were more complex and therefore increased participants’ perceptual load, leading to a larger effect. In contrast, Desmarais et al. [8] observed similar effects for both abstract and concrete stimuli: the type of stimulus did not impact how often participants responded ‘visual only’ when presented with an audiovisual stimulus. However, their participants did respond more slowly and less accurately when concrete stimuli were used. These authors suggested that an important difference between abstract and concrete stimuli is the correspondence between the auditory and visual components of the stimuli.

Indeed, when an audiovisual stimulus is made of the picture of a cat and the sound of a cat, there is correspondence between the two sensory elements: both refer to the same concept. In contrast, when an audiovisual stimulus is made of the symbol ‘Ͼ’ and a 440 Hz tone, there is no correspondence between the two sensory elements. When concrete stimuli are presented, the auditory and the visual stimuli arguably activate the same concept in memory, leading to a single multisensory perceptual event. Koppen and Spence [12] studied this phenomenon in the context of a temporal order judgement task and labeled it the unity effect. They observed that when one of the stimuli perceivably preceded the other, the Colavita effect disappeared. The authors concluded that the Colavita effect only occurs in the timeframe when the auditory and visual components of the stimulus are bound together into a single event. This unity effect would also explain why concrete stimuli give rise to a larger Colavita effect: they more easily give rise to a single perceptual event. If this is the case, why did Desmarais et al. [8] not observe a measurable difference between concrete and abstract stimuli? It could be because they used constant pairings: the same abstract visual stimulus was constantly paired with the same abstract auditory stimulus. This stability through repeated pairings can lead to an association in memory (see James and Gauthier [13] for example and Murray and Shams [14] for a review) – after a few pairings the two elements might bind more easily and give rise to a single perceptual event.

One study has looked at whether the correspondence between the identity of the auditory and visual components of audiovisual stimuli impacts the Colavita effect. Koppen et al. [11] asked participants to complete a Colavita task and used either two animal stimuli (cat and dog in Experiment 1), forty animal stimuli (Experiment 2) or speech stimuli (syllables like ‘mo,’ Experiment 3) and reported that at no point did the correspondence between the stimuli impact the magnitude of the Colavita effect. However, the correspondence between the visual and auditory stimuli did impact reaction time and accuracy – which are the same observations reported by Desmarais et al. [8] when comparing participants’ performance between abstract and concrete stimuli. Other researchers had similar findings at the neural level, but not at the behavioral level, when asking participants to complete a Colavita task with stimuli that were synesthetically congruent (i.e., large visual stimulus with low-pitched tone; small visual stimulus with high-pitched tone) or incongruent (i.e., large visual stimulus with high-pitched tone; small visual stimulus with low-pitched tone) [15].

In contrast, Stubblefield et al. [16] observed a facilitation effect from congruent stimuli in a multisensory detection task. Their participants were asked to respond when they detected a specific target presented either visually or auditorily. They responded faster and more accurately when both the visual and auditory component of stimuli belonged to the target (e.g., the picture and sound of a cat) compared to when one of the components belonged to a distracter (the picture of a cat but the sound of a cow). This was particularly pronounced when the distracter was in the visual modality: if an auditory target was paired with a visual distracter, participants failed to detect the auditory target more often. However, asking participants to respond to specific targets changes the nature of the task: it transforms a modality detection task (report the modality of stimuli) into an identification (or object recognition) task for which it is not surprising to observe semantic effects, as these have been well documented [17–19].

The observation that semantic congruence does not impact traditional Colavita tasks (as opposed to those requiring the identification of a target) is surprising because semantic congruence has been shown to impact audiovisual integration in a temporal order judgement task (see Vatakis & Spence [20], Vatakis & Spence [21] for a similar audiovisual task that is not impacted by semantics, and Vatakis et al. [22] for an audiovisual integration task comparing speech stimuli with non-human vocalizations), and in a feature discrimination task [23]. Semantic congruence between audiovisual components is also known to impact endogenous attention [24] and incongruence between visual elements in a change detection task has been shown to attract attention before detection occurs [25]. Recently, Kvasova et al. [26] have shown that the impact of crossmodal congruence on orienting may depend on task characteristics. They conducted a series of three search task experiments manipulating perceptual load and task relevance and found that relevant auditory stimuli generally accelerated search times but only in low-load conditions. The authors concluded that the automaticity of crossmodal semantic congruency may depend on attentional load. There are therefore numerous situations where audiovisual congruence impacts performance.

Stekelenburg and Keetels [15] suggested that the reason audiovisual congruence did not impact the Colavita effect is that the congruence is detected at a stage that occurs later than the Colavita effect. These authors investigated synesthetic associations using a single circle and two tones – a limited number of stimuli. Koppen et al. [11] used up to forty different concrete stimuli, but though they reported that all participants could recognize the cat and the dog (the only two stimuli used in their first experiment) in both modalities, it is not clear whether participants in the second experiment could also recognize all forty stimuli used. If participants could not recognize the various stimuli by sound, we would not expect semantic congruence to have an impact on performance: the lack of recognition would essentially nullify the difference between semantically congruent and incongruent trials. Indeed, in an attempt to select stimuli that could be recognized auditorily by participants, pilot testing in our lab revealed that participants performed quite poorly when attempting to recognize various objects, including animals, tools, and musical instruments, based on brief sounds. This is consistent with Stubblefield et al.’s [16] report that they removed a number of potential sound stimuli that were confused with the target stimuli.

One solution to the possible lack of recognition of sound stimuli would be to use spoken words. Koppen et al. [11] reported that the correspondence between the auditory and visual components of syllables (‘mo’ and ‘da’) did not influence the Colavita effect. However, they only used two stimuli, and the correspondence between a sound and lip movement might relate more to sensorimotor representations than semantic representations and the activation of concepts. One possible solution is to use the names of the stimuli instead of their sounds – the presentation of both objects/pictures and words have been shown to activate semantic representations [27]. In three experiments, we evaluated whether we could obtain a Colavita effect using words as auditory stimuli (EXP1). We then tested whether the semantic correspondence between the auditory and visual components of the stimuli impacted the Colavita effect using animal stimuli (EXP2). Finally, we evaluated whether the impact of incongruent information was different if the auditory and visual stimuli came from the same category (animals) or different categories (animals – musical instruments) (EXP3).

The goal of Experiment 1 was to demonstrate that we can obtain a reliable Colavita effect (more ‘visual-only’ response than ‘auditory-only’ responses when bimodal trials are presented) using words as the auditory stimuli. Spoken words might have a stronger correspondence with their written counterpart than with an image – we therefore also used a task where the visual stimuli were words instead of pictures. Participants were presented with visual, auditory, and audiovisual stimuli and asked to identify the modality of stimuli in three tasks: one that used pictures and sounds (a traditional Colavita task), one that used pictures and spoken words, and one that used printed words and spoken words. We hypothesized that across all three tasks, when bimodal trials were presented, participants would respond that stimuli were ‘visual-only’ more often than ‘auditory-only.’ We also hypothesized that reaction times in response to visual stimuli may be faster when words are used as visual stimuli – and that using words as visual stimuli may also lead to a greater number of ‘visual-only’ responses. As we mentioned above, the visual form of words may be more strongly linked to their sound, increasing the unity effect [11]. Chainey and Humphreys [28] have also shown that responses are faster in response to words than in response to objects in a variety of tasks. This is relevant because faster response times for responding to visual information seem to be associated with the propensity to produce ‘visual-only’ responses [29].

## 2. Experiment 1

### 2.1. Materials & methods

All of our studies were reviewed and approved by Mount Allison University’s research ethics board. Recruitment began November 16, 2022 (for Experiment 1), and ended February 28, 2025 (For Experiment 3). All participants in all three studies provided written consent. We expected our manipulation to impact the pattern of errors in response to bimodal trials; we therefore used a criterion based on the overall number of bimodal errors to determine sample size. Data were collected until we had clear support for an effect of stimulus modality where there were more errors for bimodal trials than for unimodal trials.

#### 2.1.1. Participants.

Sixty-nine participants (mean age = 19.07 years, SD = 2.28; 17 identified as male, 50 identified as female, and 2 identified as other) were recruited from Introductory Psychology courses at Mount Allison University. All participants had normal or corrected to normal vision and hearing. All participants were studying at an English-speaking institution and therefore should have a level of understanding of the English language that allows them to function in university. We therefore did not exclude participants whose first language was not English, and our sample included individuals for whom English was a second language (ESL). In the Colavita task, participants report the modality of stimuli – an understanding of the words presented is not necessary. It is possible that for ESL individuals the association between an English word and a picture is weaker, but we would expect this to reduce the size of the Colavita effect – we therefore used a conservative approach and included all participants irrespective of first language. All participants received one credit toward the completion of their course.

#### 2.1.2. Materials.

We used pictures, sounds, and words referring to ten animals: cat, bear, pig, duck, dog, cow, crow, frog, sheep, horse. Visual ‘picture’ stimuli were presented at 15° of visual angle in the centre of the screen for 350 ms. Visual ‘word’ stimuli were the written names of the animals presented in Open sans font (0.1 height) in the centre of the screen for 350 ms. Auditory ‘sound’ stimuli consisted of the sounds the animals make (e.g., quack, meow) and auditory ‘name’ stimuli consisted of the names of the animals (e.g., “duck,” “cat”) – all auditory stimuli were presented for 350ms at 65dB through speakers at both sides of the computer screen, with the sounds starting immediately at the onset of the sound file. Bimodal stimuli consisted of either the picture of an animal with its corresponding sound (e.g., picture of a cat with “meow”), the picture of an animal with its corresponding name (e.g., picture of a cat with “cat”), or a word with its corresponding name (e.g., the word cat with “cat”). The experiment was run on PsychoPy [30].

#### 2.1.3. Procedure.

Participants were seated at arm’s length from the monitor. Each trial started with a fixation point in the center of the screen for 250ms, followed by a blank screen for 1,400−1,700ms, and then a visual, auditory, or bimodal stimulus for 350ms. Participants reported each stimulus’ modality by pressing the keyboard letters ‘V’, ‘B’, and ‘N’ as quickly as possible using their dominant hand. The three keys were covered with the labels ‘V’ for visual, ‘B’ for bimodal, and ‘A’ for auditory and the order of the three keys was counterbalanced across participants. Each of the 10 stimuli were presented 10 times as visual stimuli, 10 times as auditory stimuli, and 5 times as bimodal stimuli. This resulted in a total of 100 visual trials, 100 auditory trials, and 50 bimodal trials (250 trials in total) per task. Participants completed one task where the visual stimuli consisted of pictures and the auditory stimuli consisted of animal sounds (picture-sound task), one task where the visual stimuli consisted of pictures of animals and auditory stimuli consisted of the names of animals (the picture-name task), and one task where the visual stimuli were words and the auditory stimuli were the names of animals (the word-name task). The order of the three tasks was counterbalanced between participants and testing took a total of approximately 45 minutes.

### 2.2. Results and discussion

The data of two participants was removed because they did not complete all tasks or did not follow instructions. All analyses used the default priors for JASP [31].

#### 2.2.1. Reaction time.

Reaction times (RT) to trials where the response was correct were trimmed recursively at 3 SDs, resulting in an exclusion of 4.35% of trials. We entered the data in a 3 (task) x 3 (stimulus modality) repeated measures Bayesian analysis of variance using JASP. The strongest model included a main effect of task, a main effect of stimulus modality, and an interaction between the two variables, BF10 = 3.40 x 10^19^, indicating very strong support for the alternative hypothesis. The analysis of effects confirmed that both main effects and the interaction contributed to the BF factor. Main effects were analyzed using post-hoc non-directional Bayesian t-tests. Generally, participants responded faster when responding to pictures/sounds (mean RT = 836 ms) than when responding to pictures/names (mean RT = 888 ms), BF10 = 1.38 x 10^4^; or when responding to words/names (mean RT = 933 ms), BF10 = 1.76 x 10^7^; responding to pictures/names was also faster than responding to words/names, BF10  = 27.48. Participants responded generally faster when presented with visual stimuli (mean RT = 833ms) than when presented with auditory stimuli (mean RT = 924 ms), BF10 = 1.42 x 10^17^, or bimodal stimuli (mean RT = 900 ms), BF10 = 1.38 x 10^12^; the BF 10 for the difference between RT to auditory and bimodal stimuli only provide moderate evidence for the alternative hypothesis, BF10 = 5.48.

**Fig 1:**
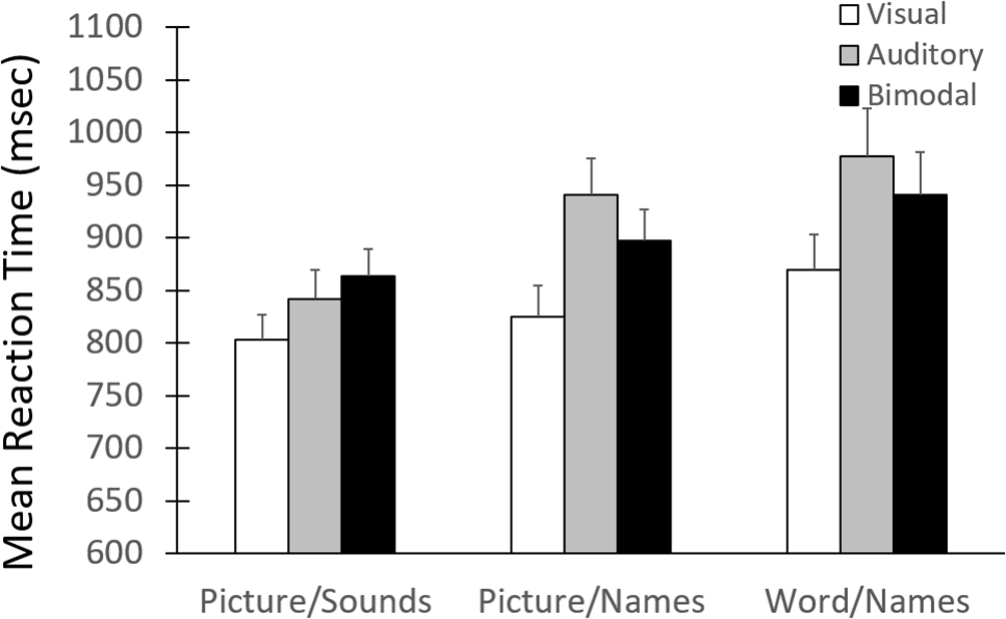
Mean RT (standard errors) for reporting the modality of visual, auditory, and bimodal stimuli when presented with pictures/sounds, pictures/names, and words/names in Experiment 1.

We analyzed the interaction by performing non-directional post-hoc paired samples t-tests comparing RT to the different modalities within each task (the results are presented in Fig 1). When participants responded to pictures and sounds, participants responded to visual stimuli (mean RT = 804 ms) faster than to auditory stimuli (mean RT = 842 ms), BF10 = 13.75, and to bimodal stimuli (mean RT = 863 ms), BF10 = 3.65 x 10^4^. The RTs in response to auditory and bimodal stimuli did not differ, BF10 = 1.02. When participants responded to pictures and names, participants responded to visual stimuli (mean RT = 825 ms) faster than to auditory stimuli (mean RT = 941 ms), BF10 = 5.32 x 10^12^, and to bimodal stimuli (mean RT = 897 ms), BF10 = 2.57 x 10^7^. Participants also responded faster in response to bimodal stimuli than auditory stimuli, BF10 = 12.15. Finally, when participants responded to words and names, participants responded to visual stimuli (mean RT = 870 ms) faster than to auditory stimuli (mean RT = 988 ms), BF10 = 1.69 x 10^5^, and to bimodal stimuli (mean RT = 941 ms), BF 10 = 54.75. Again, participants also responded faster in response to bimodal stimuli than auditory stimuli, BF10 = 9.88. The interaction therefore seems to stem from slower RT to auditory stimuli – compared to bimodal stimuli – when animal names are used instead of sounds.

#### 2.2.2. Error rates.

We entered participants’ proportion of errors for specific experimental conditions in a 3 (task) x 3 (stimulus modality) repeated measures Bayesian analysis of variance using JASP. The strongest model only included a main effect of task and a main effect of stimulus modality, BF10 = 1.54 x 10^22^, indicating very strong support for the alternative hypothesis. The analysis of effects confirmed that only these two variables contributed to the BF factor. Main effects were analyzed using post-hoc non-directional Bayesian t-tests. Generally, participants produced fewer errors when presented with pictures and sounds (mean error rate = .04) or pictures and names (mean error rate = .04) than when presented with words and names (mean error rate = .06), BF = 426.53 and 181.08 respectively. The error rates did not differ between the picture/sound and picture/names tasks, BF01 = 12.82 – indicating strong support for the null hypothesis. Participants also produced more errors when presented with bimodal stimuli (mean error rate = .08) than when presented with visual stimuli (mean error rate = .03, BF10 = 6.98 x 10^22^) or when presented with auditory stimuli (mean error rate = .02, BF10 = 9.65 x 10^26^. Participants also produced more errors in response to visual stimuli than in response to auditory stimuli, BF 10 = 937.75.

#### 2.2.3. Colavita effect.

Finally, we separated the errors made in response to bimodal trials into ‘visual-only’ and ‘auditory-only’ responses and entered the data in a 3 (task) x 2 (error type) within subjects Bayesian ANOVA. The strongest model only included a main effect of error type, BF10 = 1.30 x 10^5^, providing very strong evidence for the alternative hypothesis. The analysis of effects confirmed that only error type contributed to the BF factor. Across all tasks, when participants were presented with a bimodal trial, they responded ‘visual-only’ (mean error rate = .06) more often than ‘auditory-only’ (mean error rate = .02).

As predicted, there were more ‘visual-only’ than ‘auditory-only’ errors across all three tasks. Importantly, there was no interaction between the task used and the frequency of ‘visual-only’ or ‘auditory-only’ responses, which means that a Colavita effect can be obtained with words as the auditory stimulus. This can facilitate the investigation of semantic correspondence because we can use words as sound stimuli – we expect these to be more easily recognizable than the sounds of objects.

Contrary to our prediction, response times were not faster when words were used as visual stimuli – nor did visually-presented words generate more ‘visual-only’ responses. This might have occurred because the Colavita task does not require the identification of stimuli. Though multiple explanations have been suggested for the Colavita effect (see Spence [32] for a review), the faster response times for responding to visual information compared to auditory information seem to be particularly important: faster responses to visual information have been associated with a greater number of ‘visual-only’ responses [29]. We therefore recruited a second group of participants that have been shown to have faster RT: athletes. The frequent cardiovascular exercise that athletes engage in has been repeatedly associated with faster responses on cognitive tasks like the Stroop task [33], visuo-spatial processing [34], a flanker task [35], an oddball task [36] and importantly, visual detection [37,38]. We can therefore expect athletes to have faster responses times than nonathletes during our Colavita tasks. To confirm our sample size and make sure we would have enough participants to detect the Colavita effect, we calculated the effect size for the Colavita effect observed in Experiment 1: *partial η*^*2*^ = .342 (power was near 1.00). Given the effect size we used the G-power software to confirm that a minimum sample size of 30 per group would be sufficient to detect the effect with 99% power, but aimed to recruit a minimum of 45 participants per group to compensate for the unknown effect size for semantic correspondence.

The goal of experiment 2 was to demonstrate that the semantic correspondence between the visual and auditory components of audiovisual stimuli impact the Colavita effect. Our participants (athletes and nonathletes) completed two Colavita tasks: one with pictures and sounds, and another with pictures and names. The lack of interaction with task in Experiment 1 led us to drop the task containing words and names as it was redundant with the other two – we maintained the Colavita task containing pictures and sounds for comparison. Participants were presented with visual, auditory, and audiovisual stimuli and asked to report the modality of stimuli. Crucially, half of the bimodal trials contained congruent elements, while the other half contained incongruent elements. We hypothesized that there will be more ‘visual-only’ than ‘auditory-only’ responses during bimodal trials (a Colavita effect), and that this effect will be greater during congruent trials than during incongruent trials. Furthermore, we expect athletes to have faster response times and a greater number of ‘visual-only’ responses than nonathletes.

## 3. Experiment 2

### 3.1. Materials & methods

#### 3.1.1. Participants.

One-hundred and four participants (mean age = 19.25 years, SD = 3.18; 87 participants identified as female, 12 identified as male, and 2 participants did not specify gender) were recruited from Introductory Psychology courses and various sports teams at Mount Allison University. Three participants were removed from the sample due to incomplete data or high error rate, leaving only 101 participants. The sample consisted of 46 non-athletes (six identified as male), and 55 athletes (six identified as male). In this series of studies, the term athlete referred to any participant who self-reported as being an athlete member of a sport team (individual or team sport), and the term non-athlete referred to all other participants. All participants reported having normal or corrected to normal vision and hearing. The students recruited from Introductory Psychology classes received an incentive of 1 credit toward the completion of their course while those recruited from sports teams received twelve dollars in compensation for their participation.

#### 3.1.2. Materials.

Participants completed a demographics questionnaire assessing characteristics including age, gender, handedness, and whether they were a student-athlete, and in which sport. The latter was used to classify participants into athletic/non-athletic groups. Individuals who reported being part of a sports team (whether a club or their university’s varsity team) within the last year were considered athletes – it is therefore possible that athletic individuals were included in the ‘non-athlete’ group.

The visual and auditory stimuli were the same ten pictures, ten sounds, and ten names of animals used in Experiment 1. Importantly, the bimodal stimuli consisted of congruent and incongruent stimuli. For congruent stimuli, the auditory and visual component associated with the same animal were presented simultaneously (e.g., the sound of a dog with the picture of a dog, or the auditory word “dog” with the picture of a dog). For incongruent stimuli, the auditory and visual component associated with two different animals were presented simultaneously (e.g., the sound of a dog with the picture of a cat, or the auditory word “dog” with the picture of a cat). For a given participant, the same incongruent stimuli were used through the experiment. There were seven stimulus groups, and each had a different set of incongruent combinations. For example, any participants that were assigned to group ‘A’ received a set combination of incongruent stimuli (e.g., the picture of a dog was always paired with the sound of a cat) while group ‘B’ received a different set of combinations of incongruent stimuli (e.g., the picture of a dog was always paired with the sound of a duck). The experiment was run on PsychoPy [30].

#### 3.1.3. Procedure.

The procedure was identical to that described for Experiment 1, except for the number of stimuli and that there was no task involving visually presented words. Each of the 10 stimuli was presented 16 times as visual stimuli, 16 times as auditory stimuli and 8 times as bimodal stimuli (4 congruent trials and 4 incongruent trials). This resulted in 400 trials per task, and each participant completed two tasks, one with animal pictures and sounds and one with animal pictures and names, which took about 45 minutes in total. The order of the tasks was counterbalanced across participants.

### 3.2. Results and discussion

#### 3.2.1. Reaction times.

RTs were trimmed recursively at 3 SDs, which resulted in the removal of 4.58% of trials. The data were entered in a 2 (participant type: athlete or non-athletes) x 3 (trial modality: visual, auditory, or bimodal) x 2 (task: picture/sounds or picture/words) mixed design Bayesian ANOVA where the only between factor was participant type. Only RTs to congruent trials were included as unimodal trials could not be incongruent. The strongest model included main effects of task type, trial modality, and participant type, along with two interactions: one between task type and trial modality, and the other between participant type and task, BF10 = 3.49 x 10^36^, providing very strong support for the alternative hypothesis. The analysis of effects confirmed that the main effects and interaction between task and stimulus modality contributed to the BF factor, but the contribution of the interaction between participant type and task only contributed moderately to the BF factor.

Generally, athletes (mean RT = 775 ms) responded faster than non-athletes (mean RT = 895 ms), and participants responded faster when presented with pictures and sounds (mean RT = 822 ms) than when presented with pictures and names (mean RT = 848 ms). The mean effect of stimulus modality was analyzed using post-hoc paired non-directional Bayesian t-tests. Generally, participants responded faster when presented with visual stimuli (mean RT = 768 ms) than when presented with auditory stimuli (mean RT = 858 ms) or bimodal stimuli (mean RT = 879 ms), BF10 = 1.87 x 10^25^ and 1.40 x 10^31^ respectively. Participants also responded faster when presented with auditory stimuli than when presented with bimodal stimuli, BF10 = 12.34.

**Fig 2:**
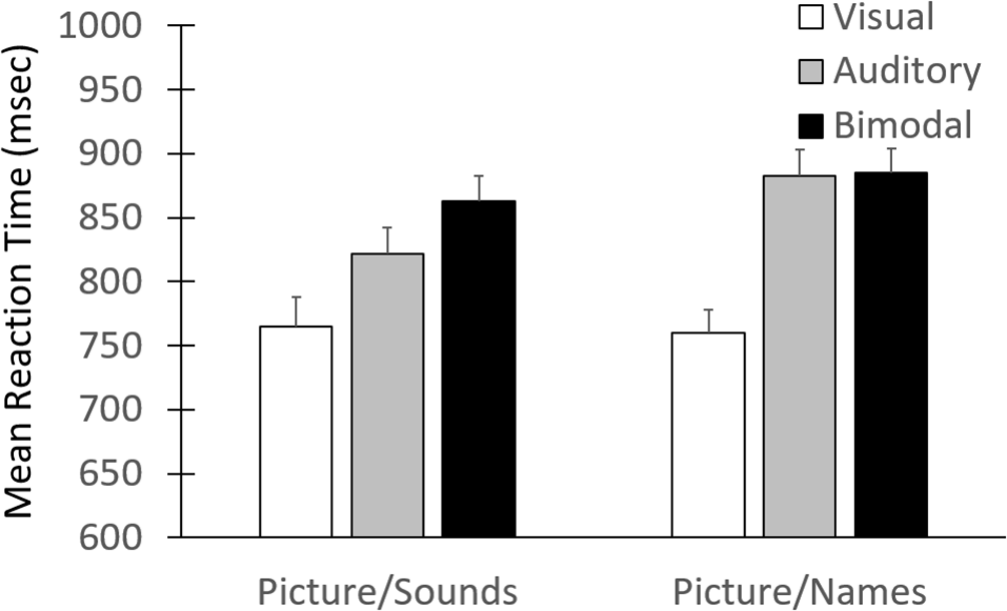
Mean RT(and standard error) for responding to visual, auditory, and bimodal stimuli when presented with pictures and sounds or pictures and names in Experiment 2.

The interactions were analyzed using post-hoc paired non-directional Bayesian t-tests for the different stimulus modalities within each task (the results are presented in Fig 2). When participants responded to pictures and sounds, they responded faster when presented with visual stimuli (mean RT = 765 ms) than when presented with auditory stimuli (mean RT = 822 ms), or bimodal stimuli (mean RT = 863 ms), BF10 = 5.23 x 10^5^ and 1.06 x 10^10^, respectively. Participants also responded faster when presented with auditory stimuli than when presented with bimodal stimuli, BF10 = 2.38 x 10^3^. When participants responded to pictures and names, they responded faster when presented with visual stimuli (mean RT = 760 ms) than when presented with auditory stimuli (mean RT = 883 ms) or bimodal stimuli (mean RT = 885 ms), BF10 = 1.49 x 10^21^ and 2.25 x 10^22^ respectively. However, response times did not differ between responding to auditory and bimodal stimuli, BF01 = 8.88 – indicating strong support for the null hypothesis.

The interaction between task and participant type did not contribute much to the model. Athletes’ RTs when presented with pictures and sounds (mean RT = 780 ms) was similar to their RTs when presented with pictures and names (mean RT = 809 ms), and the BF factor did not provide evidence towards either the null or alternative hypothesis, BF10 = 1.03. Similarly, nonathletes’ RTs when presented with pictures and sounds (mean RT = 901 ms) was similar to their RTs to pictures and words (mean RT = 920 ms), BF01 = 5.22, providing moderate evidence towards the null hypothesis.

#### 3.2.2. Error rates.

Error rates were entered in a 2 (participant type) x 3 (trial modality) x 2 (task) mixed design Bayesian ANOVA where the only between factor was participant type. Only error rates for congruent bimodal trials were included because the unimodal trials could not be incongruent. The strongest model only included a main effect of trial modality, BF10 = 4.16 x 10^30^, providing very strong evidence for the alternative hypothesis. The analysis of effects confirmed that only this variable contributed to the BF factor. This main effect was analyzed using post-hoc paired non-directional Bayesian t-tests. Generally, participants produced the fewest errors when presented with auditory stimuli (mean error rate = .02) when compared to visual stimuli (mean error rate = .03) or bimodal stimuli (mean error rate = .15), BF10 = 986.00 and BF10 = 2.41 x 10^29^, respectively. Participants also produced fewer errors when presented with visual stimuli than when presented with bimodal stimuli, BF10 = 1.05 x 10^28^.

#### 3.2.3. Colavita effect.

Finally, we separated the errors made in response to bimodal trials into ‘visual-only’ and ‘auditory-only’ responses and entered the data in a 2 (task) x 2 (error type) x (congruence) x 2 (participant type) mixed design Bayesian ANOVA where the only between factor was the type of participant. The strongest model included main effects of task, congruence, and response type, as well as interactions between task and response type, and between congruence and response type, BF10 = 3.50 x 10^14^, providing very strong evidence for the alternative hypothesis. Generally, participants produced fewer errors when presented with pictures and sound (mean error rate = .04) than when presented with pictures and names (mean error rate = .04), and they produced more errors when audiovisual stimuli were congruent (mean error rates = .05) than when audiovisual stimuli were incongruent (mean error rate = .03); they also responded ‘visual-only’ (mean error rate = .06) more often than ‘auditory-only’ (mean error rate = .02).

**Fig 3:**
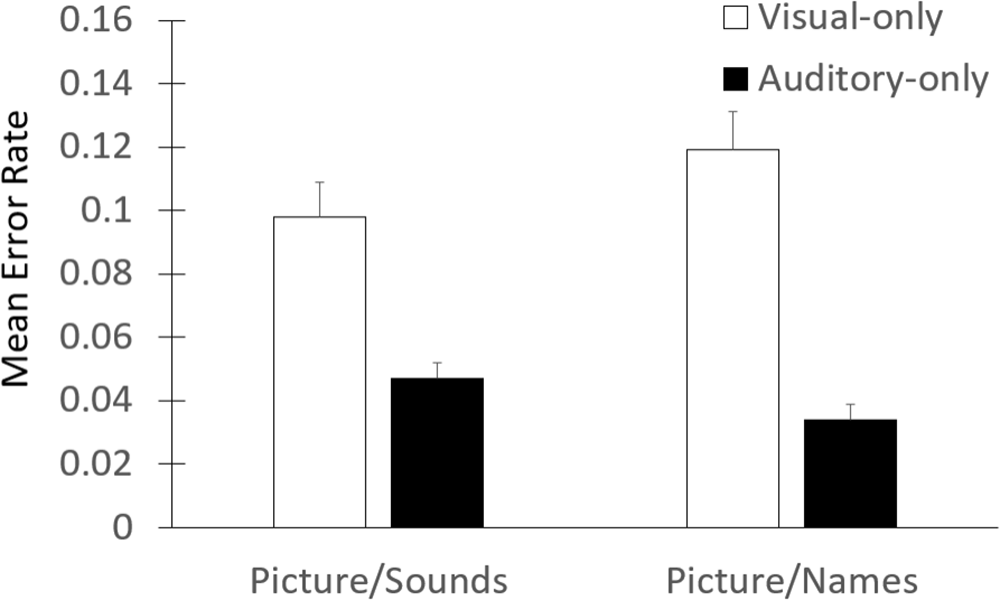
Mean error rates (and standard error) corresponding to ‘visual-only’ and ‘auditory-only’ responses when responding to pictures/sounds, and pictures/names in Experiment 2.

**Fig 4:**
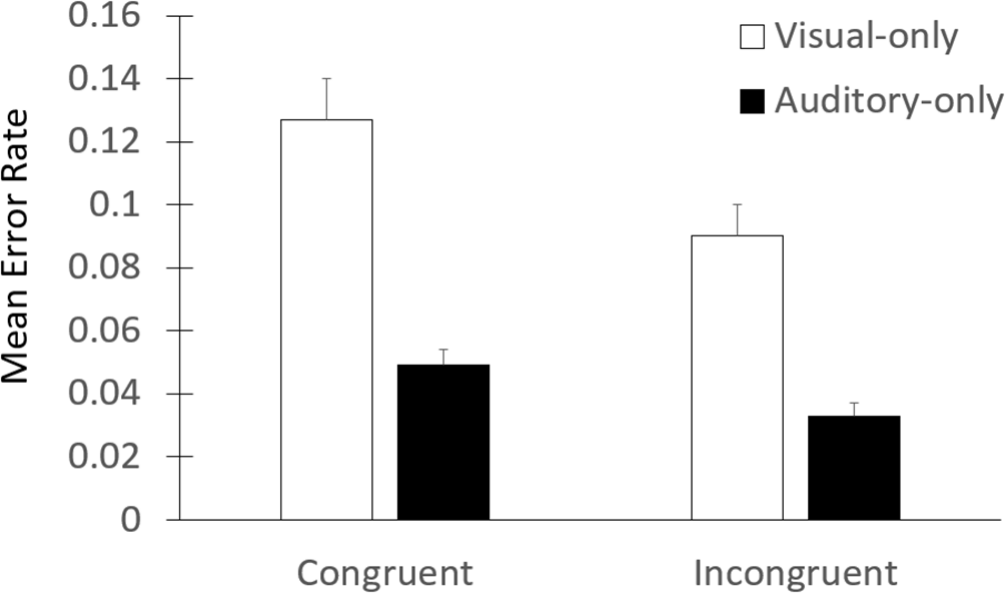
Mean error rates (and standard error) corresponding to ‘visual-only’ and ‘auditory-only’ responses when responding to congruent and incongruent stimuli in Experiment 2.

We analyzed the interaction between task and response type using directional paired post hoc Bayesian t-tests. When participants responded to pictures and sounds, there were more ‘visual-only’ responses (mean error rate = .10) than ‘auditory-only’ responses (mean error rate = .05), BF10 = 1.77 x 10^4^, a difference of  .05. When participants responded to pictures and names, there were more ‘visual-only’ responses (mean error rate = .12) than ‘auditory-only’ responses (mean error rate = .03), BF10 = 1.35 x 10^9^, a difference of  .09 – the interaction therefore stemmed from a larger difference when pictures and names were presented (see Fig 3). We analyzed the interaction between congruence and response type using planned directional t-tests. When participants responded to congruent trials, there were more ‘visual-only’ responses (mean error rate = .13) than ‘auditory-only’ responses (mean error rate = .05), BF10 = 5.51 x 10^6^, a difference of  .078. When participants responded to incongruent trials, there were more ‘visual-only’ responses (mean error rate = .10) than ‘auditory-only’ responses (mean error rate = .03), BF10 = 1.10 x 10^7^, a difference of  .06 (see Fig 4). The interaction therefore stemmed from a larger difference between visual-only and auditory-only responses (a larger Colavita effect) when stimuli were congruent.

As predicted, athletes were faster than nonathletes. This is unlikely to be a speed/accuracy tradeoff as the athletes did not produce more errors than the nonathletes. These faster RTs were not, however, associated with a larger Colavita effect. It is possible that despite practicing a sport, our athletes were not more fit than our nonathletes. Therefore, in Experiment 3, we will measure resting heart rate in order to confirm the physiological impact of the athletes’ regular exercise.

Importantly, as predicted, the Colavita effect was larger when the auditory and visual component of stimuli were congruent than when they were incongruent. This evidence contradicts Koppen et al. [11] but would align with studies showing impacts from semantic correspondence on other cognitive tasks [22,39,40]. Possibly, the congruent auditory and visual components were perceived as belonging to the same item and were therefore bound more easily. If this is the case, we might see a gradation within the Colavita effect that would depend on the degree of correspondence between the visual and the auditory component of bimodal stimuli. When the visual and auditory component refer to the same concepts (the picture of a cat with the sound of a cat), then the two stimuli are easily bound and produce the most ‘visual-only’ errors. When the two stimuli come from different items from the same category (the picture of a cat and the sound of a dog), then the two stimuli are less easily bound and produce fewer ‘visual-only’ errors. If the two stimuli came from items from different categories (the picture of a cat and the sound of a horn), then we would expect even less binding and even fewer ‘visual-only’ errors.

The aim of Experiment 3 was to evaluate whether the impact of semantic correspondence on the Colavita effect would be impacted by the degree of correspondence between the visual and auditory component of bimodal stimuli. Two groups of participants (athletes and nonathletes) completed Colavita tasks where the bimodal stimuli consisted of components that were associated with either the same item, two items from the same category (incongruent-within) or two items from different categories (incongruent-between). We hypothesized that the size of the Colavita effect would depend on the degree of correspondence between the auditory and the visual component bimodal stimuli. We expected the most ‘visual-only’ responses when bimodal stimuli were congruent, and the fewest ‘visual-only’ responses when bimodal stimuli were incongruent with components coming from different categories.

## 4. Experiment 3

### 4.1. Materials & methods

#### 4.1.1. Participants.

One hundred and four students enrolled at Mount Allison University participated in this study. Fifty were varsity or club athletes and 54 were non-athletes (were not part of an athletic team). Participants’ mean age was 19.15 years (*SD* = 1.61) and 73 identified as female, 28 identified as male (14 were athletes) and 3 identified as other. They were given either one course credit or $14 per hour as an incentive for participating in the study (these participants were tested in a different academic year than participants in Experiment 1 and Experiment 2, after participant compensation was increased).

#### 4.1.2. Materials.

Baseline heart rate and oxygen saturation levels were measured using a pulse oximeter.

The stimuli consisted of 5 of the animals used in the previous experiments (cat, dog, pig, cow, and horse) and 5 musical instruments (drum, harp, flute, tuba, and guitar). The new stimuli were similar in size (for visual stimuli) and loudness (for auditory stimuli) as the animal stimuli. As in Experiment 2, congruent bimodal stimuli comprised a visual and auditory component that referred to the same concept (the image and sound of a cat) and incongruent stimuli comprised visual and auditory components that referred to different concepts. However, there were two types of incongruent stimuli. Within category incongruent stimuli consisted of a visual and auditory component from the same category (e.g., the image of a cat paired with the sound of a dog). Between category incongruent stimuli consisted of a visual and auditory component from different categories (e.g., the image of a cat paired with the sound of a drum). For a given participant, a specific visual stimulus was always paired with the same incongruent sound, and this was counterbalanced between participants.

#### 4.1.3. Procedure.

The procedure was identical to that of Experiment 2 except for the number of stimuli. Each of the ten stimuli was presented 16 times visually, 16 times auditorily, and eight times bimodally (four congruent trials, two incongruent within trials, and two incongruent between trials), for a total of 40 times in each task. Therefore, there were 160 visual trials, 160 auditory trials, and 80 bimodal trials (40 congruent, 20 incongruent within, and 20 incongruent between), for a total of 400 trials per task. Participants took approximately 45 minutes to complete the study.

### 4.2. Results and discussion

A planned Bayesian independent samples t-test confirmed that the heart rate of athletes (*M* = 73.7 bpm) was lower than the heart rate of nonathletes (*M* = 87.4 bpm), BF10 = 2631.00.

#### 4.2.1. Reaction times.

**Fig 5:**
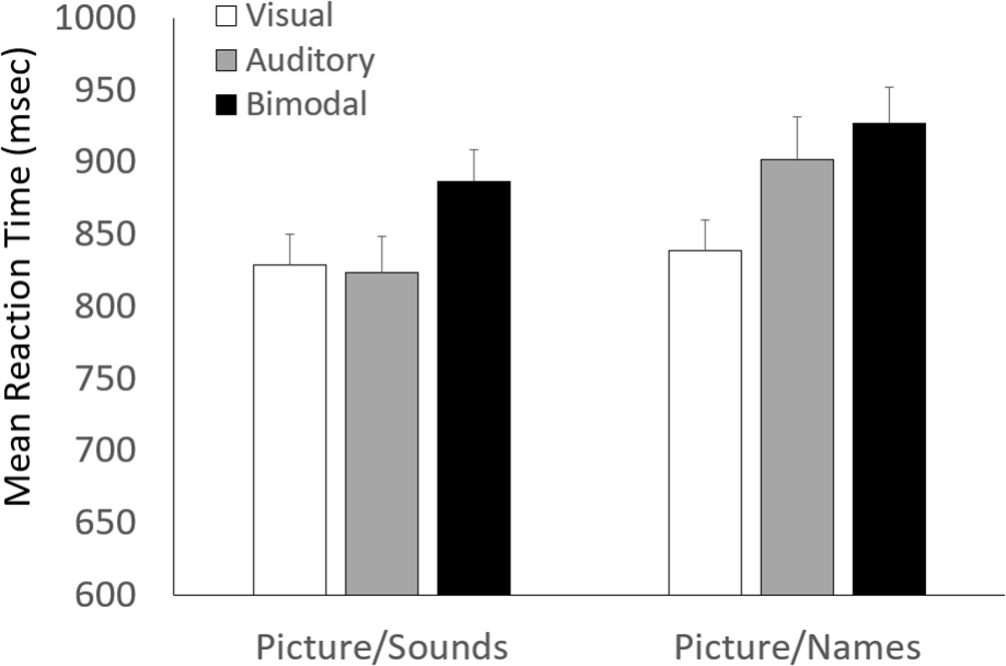
Mean RTs (and standard error) when responding to auditory, visual, and bimodal stimuli when responding to pictures/sounds, and pictures/names in Experiment 3.

RT for correct trials were trimmed recursively at three SDs, and this removed 4.80% of trials. The data was entered in a 2 (participant type) x 2 (task) x 3 (modality) Bayesian mixed measures ANOVA where participant type was the only between subjects factor. Only RTs for congruent bimodal trials were included because the unimodal trials could not be incongruent. The strongest model included main effects of participant type, task, and modality, and an interaction between task and modality, BF10 = 8.90 x 10^10^, providing very strong support for the alternative hypothesis. The analysis of effects confirmed that only those three effects / interaction contributed to the model. Generally, athletes (mean RT = 807 msec) responded faster than nonathletes (mean RT = 925 msec), and all participants responded faster when presented with pictures and sounds (mean RT = 845 msec) than when presented with pictures and words (mean RT = 887 msec). The pattern of RTs in responses to different stimulus modalities depended on the task (see Fig 5). When presented with pictures and sounds, RTs were slower in response to bimodal stimuli (mean RT = 887 msec) than in response to visual stimuli (mean RT = 829 msec) or auditory stimuli (mean RT = 824 msec), BF10 = 8.31 x 10^3^ and BF10 = 1.17 x 10^4^ respectively. RTs to auditory and visual stimuli did not differ, BF01 = 8.70. However, when presented with pictures and words, RTs were slower in response to bimodal stimuli (mean RT = 927 msec) than in response to visual stimuli (mean RT = 839 msec) but not slower than in response to auditory stimuli (mean RT = 902 msec), BF10 = 1.41 x 10^8^ and BF01 = 2.22 respectively. RTs were also faster in response to visual stimuli than in response to auditory stimuli, BF10 = 109.60.

#### 4.2.2. Errors rates.

Error rates were entered in a 2 (participant type) x 2 (task) x 3 (modality) Bayesian mixed measures ANOVA where participant type was the only between subjects factor. Only error rates for congruent bimodal trials were included because the unimodal trials could not be incongruent. The strongest model included main effects of participant type and modality as well as an interaction between these two factors, BF10 = 4.51 x 10^27^, providing very strong evidence for the model. Generally, athletes produced more errors (mean error rate = .04) than non-athletes (mean error rate = .03) but the analysis of effects only yielded anecdotal evidence for this factor, BF10 = 0.75. The individual BF for the interaction was likewise anecdotal, BF10 = 1.29, and we therefore focused on the main effect of modality, for which the individual BF10 = Ꚙ. Participants produced more errors in response to bimodal stimuli (mean error rate = .08 errors) than in response to auditory stimuli (mean error rate = .02 errors) or visual stimuli (mean error rate = .02 errors), BF10 = 1.02 x 10^14^ and BF10 = 1.36 x 10^14^ respectively. The mean error rates in response to auditory and visual stimuli did not differ, BF10 = 1.83.

#### 4.2.3. Colavita effect.

**Fig 6:**
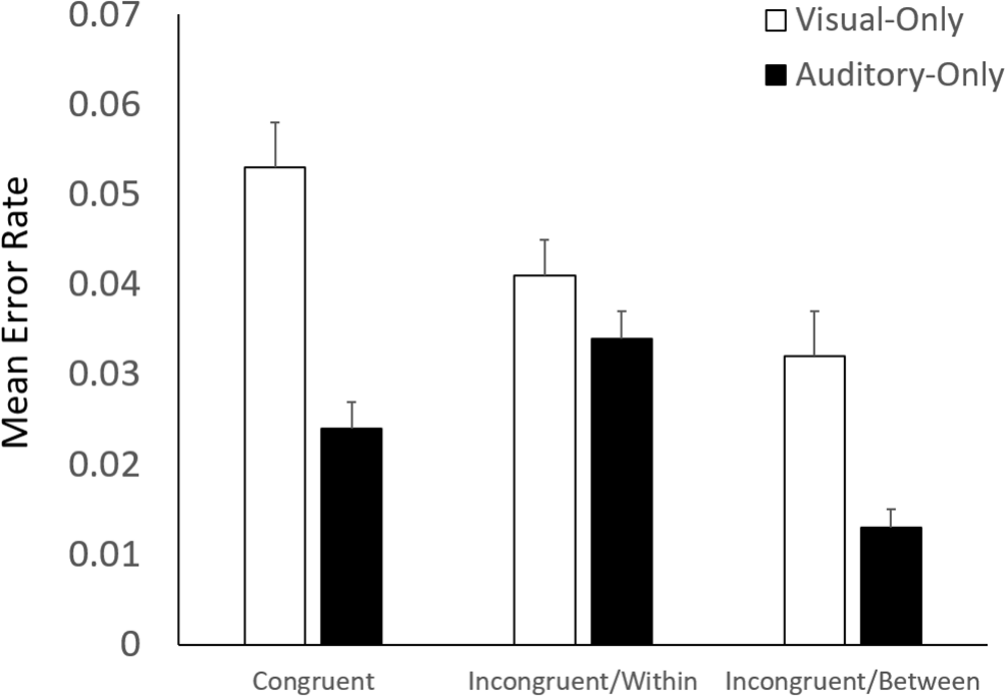
Mean rate of ‘visual-only’ and ‘auditory-only’ responses (and standard error) when the bimodal stimuli are congruent, incongruent but within category, and incongruent and between category (Experiment 3).

Finally, we separated the errors in response to bimodal trials into ‘visual-only’ and ‘auditory-only’ responses and entered the data into a 2 (participant type) x 2 (task) x 3 (semantic correspondence) x 2 (response type) Bayesian mixed-measures ANOVA where participant type was the only between-subject factor. The strongest model included a main effect of semantic correspondence, a main effect of response type, and an interaction between these two factors, BF10 = 2.38 x 10^13^, providing very strong evidence for the alternative hypothesis. Generally, participants produced more ‘visual-only’ responses (mean error rate = .04) than ‘auditory-only’ responses (mean error rate = .02). They also produced more errors in response to congruent trials (mean error rate = .04) than in response to incongruent within trials (mean error rate = .03) or incongruent between trials (mean error rate = .02); BF10 = 299.00 and BF10 = 2.01 x 10^9^, respectively. The two types of incongruent trials resulted in similar error rates, BF01 = 1.98, providing anecdotal support for the null hypothesis. The interaction was analyzed by conducting planned directional paired samples Bayesian t-tests comparing the rates of visual-only vs. auditory-only responses at each level of congruence (results are displayed in Fig 6). There were always more ‘visual-only’ than ‘auditory-only’ responses. The largest difference was observed when the stimuli were congruent, where the mean error rate was 0.05 for ‘visual-only’ responses and 0.02 for ‘auditory-only’ responses, BF10 = 1.216 x 10^7^. The difference between ‘visual-only (mean error rate = 0.03) and ‘auditory-only’ (mean error rate = 0.01) responses was smaller for incongruent trials composed of items from different categories, BF10 = 88.60. The difference between ‘visual-only (mean error rate = 0.04) and ‘auditory-only’ (mean error rate = 0.03) responses was smallest for incongruent trials composed of items from the same category, BF10 = 31.55.

## 5. General discussion

We aimed to evaluate whether semantic correspondence would impact the frequency of ‘visual-only’ responses when participants were presented with bimodal stimuli in the context of a Colavita task. In a first experiment, we established that the Colavita effect could be replicated with word stimuli: participants produced more ‘visual-only’ errors than ‘auditory-only’ errors when bimodal stimuli were presented; this occurred when stimuli were pictures or printed words paired with sounds or spoken words. We then manipulated semantic correspondence and observed that there were more ‘visual-only’ errors in response to bimodal stimuli when the two components of the stimuli referred to the same concept (e.g., the picture of a cat with the sound of a cat) than when the two components of the stimuli referred to different concepts (e.g., the picture of a cat with the sound of a dog).

Research on the Colavita effect has mostly focused on either abstract stimuli [e.g., 1, 8] or concrete stimuli that comprised pictures and sounds [e.g., 6, 7, 11]. Our studies were the first to use words as stimuli in this task. Using words as auditory stimuli led to slower response times when responding to auditory stimuli but did not otherwise impact the magnitude of the Colavita effect. In the case of spoken words, slower processing could have reduced the Colavita effect by reducing ‘unity’. This means that when presented with the sound “cat” and a picture of a cat, the auditory stimulus is not facilitating detection of the visual stimulus, as shown in another study of the Colavita effect [6]. However, the type of auditory stimuli used (sounds vs. spoken words) generally did not impact performance. In Experiment 2, the Colavita effect was larger – not smaller – when words were used as auditory stimuli, and this did not interact with the congruence of the audiovisual stimuli. This observation is inconsistent with the idea that word auditory stimuli would be less well connected to pictures than animal sounds. The Colavita tasks can therefore be carried out using words as either visual stimuli or sound stimuli, which opens the possibility of manipulating various kinds of conceptual information associated with concepts.

Importantly, we have shown in two experiments that manipulating the semantic correspondence of the visual and auditory components of stimuli impacts the number of ‘visual-only’ responses. Generally, there was a greater difference between the number of visual-only responses and the number of auditory-only responses when the visual and auditory component of stimuli corresponded to the same concept. This finding is in direct contrast to Koppen et al [11] who observed that semantic correspondence did not impact the Colavita effect but aligns with other studies showing the impact of semantic correspondence on perceptual tasks [22,39,40]. If concepts that are more easily bound together lead to a stronger Colavita effect [12,39], this suggests that using congruent stimuli results in stronger binding than using incongruent stimuli. Concrete stimuli activate a concept in memory; congruent stimuli would therefore activate the one concept in memory while incongruent stimuli would activate two different concepts in memory (one for the visual stimulus and another for the auditory stimulus). Sinnett et al. [6] proposed that facilitation between co-occurring auditory and visual stimuli result in the auditory component becoming redundant in the presence of the visual component. Consequently, we would expect that this facilitation of the visual component would be stronger when the auditory stimulus is referring to the same object or concept in memory as the visual stimulus. Returning to the concept of binding, if we consider the example of seeing a picture of a dog accompanied by the sound of a cat, the cat sound would not bind as easily with the picture of the dog because these two pieces don’t activate the same concept in memory. This would make it easier to perceive both the auditory and visual component because they are easier to separate. Since activation of the visual cortex can suppress that of the auditory cortex [41], it is possible that the redundancy of the auditory component during the binding process allows this suppression to be even more pronounced. The improved binding of the auditory and visual component during congruent trials would thus naturally increase suppression of activity in the auditory cortex, resulting in more ‘visual-only’ responses for congruent stimuli compared to incongruent stimuli.

Previous research has also suggested that semantic processing occurs after stimulus detection [11], however, our findings suggest that stimulus identity and semantic information can still influence early perceptual processes. This would be consistent with the findings in fMRI studies that demonstrate increased sensitivity and activation for incongruent audiovisual stimuli, as well as the activation of different cortical locations in response to congruent and incongruent stimuli [42]. This could suggest that congruent stimuli are quickly interpreted as a single percept while incongruent stimuli are interpreted as two independent objects.

Interestingly, in Experiment 3 when we used three levels of correspondence (congruent, incongruent within category, and incongruent between categories), there were more auditory-only responses than expected when the visual and auditory components of stimuli corresponded to different items from the same category. This was surprising as it was not observed in Experiment 2 when only two levels of correspondence were used (congruent or incongruent within category). Though this is potentially due to random variability, it is also possible that it is the consequence of having multiple levels of correspondence. The three levels of correspondence create degrees of similarity. During congruent bimodal trials, the auditory and visual stimuli refer to identical concepts (the picture of a cat and sound of a cat), while during incongruent within-category trials the auditory and visual stimuli refer to somewhat similar concepts (the picture of a cat and sounds of a dog both refer to animals), and during incongruent between-category trials the auditory and visual stimuli refer to distinct concepts. Research on multisensory object identification has shown that when different objects are presented (participants have to identify a visually-presented object but ignore an incongruent haptically-presented object), the similarity between the objects influences performance: similar objects produce interference while distinct objects do not [43]. It is possible that when there are multiple levels of congruence, the incongruent stimuli that refer to items from the same category generate interference. In this context, congruent stimuli would be integrated more quickly and easily, resulting in a large Colavita effect; incongruent stimuli with components coming from different categories would not be well integrated, but the discrepancy could easily be ignored (like distinct distractors in object identification studies), resulting in a smaller Colavita effect. Finally, incongruent stimuli with components coming from the same category could generate competition – resulting in an even smaller Colavita effect. This kind of modulation of the Colavita effect generated by task characteristics would be consistent with other modulations observed [8]. We should note, however, that we used the difference between the number of visual-only and auditory-only responses to evaluate the magnitude of the Colavita effect. If we were to look only at ‘visual-only’ responses the pattern of performance matches our predictions perfectly: the largest number of visual-only errors occurred for congruent bimodal trials, followed by incongruent trials where the items came from the same category, and the smallest number of visual-only errors occurred for incongruent trials where the items came from distinct categories.

Our findings include multiple observations that run contrary to evidence from another Colavita study (see Koppen & Spence [2]). In Koppen and Spence’s Experiment 3 [2] visual-only errors on bimodal trials were significantly faster than unimodal visual trials and correctly reported bimodal trials. While reviewing this finding, Spence et al. [29] conclude that ‘visual-only’ responses to bimodal trials are tightly linked to speed of response. Countering this point, we included participants that specifically differed in the speed at which they respond to stimuli – our athletes were significantly faster than our non-athletes. Despite this significant difference in response time, the athletes did not produce more ‘visual-only’ responses than non-athletes. Furthermore, we also used stimuli to which participants were slower to respond compared to other auditory stimuli: participants were slower when responding to names (e.g., “cat”) than when responding to sounds (e.g., “meow”). Despite this significant difference in response times to specific auditory stimuli, the two types of stimuli did not produce measurably different Colavita effects. Clearly, the speed at which visual stimuli are processed do not always predict the Colavita effect.

Though we have shown that semantic correspondence impacts the Colavita effect, we demonstrated this using specific stimuli and it will be important to replicate the effect using additional stimuli in order to make the findings more generalizable. It would also be useful to evaluate whether congruence impacts the auditory suppression observed by other researchers [41]. Importantly, we have shown that a Colavita effect can be obtained with verbal auditory stimuli (the names of objects), which will hopefully facilitate stimulus selection in future research.
