# Supplementary material for: Semantic congruence impacts audiovisual processing in the Colavita effect
Source: PLoS One. 2026 Jul 6;21(7):e0352876. doi: 10.1371/journal.pone.0352876 (PMC13336217; doi:10.1371/journal.pone.0352876)
Supplement: Attachment — Submitted filename: Responses to reviewers.docx [file pone.0352876.s002.docx]

Responses to reviewers

Reviewer #1

Minor point 1: l.16 ‘Robust’ – not sure about that. Often-demonstrated yes, but a very small effect,

*Response: we have removed the term ‘robust’- this has been done throughout the manuscript for consistency.*

Minor point 2: l. 18 ‘often’ – I would say ‘occasionally’

*Response: we have replaced ‘often’ with ‘occasionally’*

Minor point 3: l. 39 ‘quite robust’ – backpeddling?

*Response: we have replaced the term ‘quite robust’ with ‘reliably demonstrated effect’*

Minor point 4: l. 68 provide reference on this.

*Response: the statement is a suggestion as to what might have happened, not a reference to specific previous findings. We have nonetheless referenced a relevant past finding: “This stability through repeated pairings can lead to an association in memory (see James and Gauthier 2003 for example and Murray and Shams 2023 for a review) – after a few pairings the two elements might bind more easily and give rise to a single perceptual event.”(lines 67-69)*

Minor point 5: l. 93 Note also Vatakis, A., Ghazanfar, A., & Spence, C. (2008). Facilitation of multisensory integration by the “unity effect” reveals that speech is special. Journal of Vision, 8/9/14, 1-11. <https://doi.org/10.1167/8.9.14>.

*Response: we have added the reference.*

Minor point 6: 174. Given that p(bimodal) influences likelihood of observing the Colavita effect, is the % here quite high

*Response: the proportion of auditory, visual, and bimodal trials (20% of bimodal trials) is consistent with our past studies to produce a Colavita effect. It is also consistent with other studies of the Colavita effect. Koppen and Spence replicated the effect using 40/40/20 and 33/33/33 and reported that: “These results therefore suggest that stimulus probability and the response demands of the task do not contribute significantly to the Colavita effect”*

Minor point 7: 194 |Avoid starting sentence with ‘Also…’

*Response: we have removed ‘Also’ from the sentence.*

Minor point 8: 246 ‘could occur’ tense seems wrong

*Response: we have replaced ‘could occur’ with ‘might have occurred.’*

Minor point 9: 260’traditional Colavita task’ sounds too strong a claim

*Response: we replaced ‘traditional Colavita task’ with ‘Colavita task containing pictures and sounds’*

Minor point 10: 281-282 More details on classification helpful, and presumably an arbitraty cut-off, and that many athletes may not be part of club or team?

*Response: We clarified that all individuals that reported being part of a sports team were classified as athletes: “Individuals who reported being part of a sports team (whether a club or their university’s varsity team) within the last year were considered athletes,it is therefore possible that athletic individuals were included in the non-athlete group.”*

Minor point 11: 326 add ‘,’

*Response: a comma has been added*

Minor point 12: 391 Improve start of sentence

*Response: we have rephrased the sentence “. It is possible that despite practicing a sport, our athletes were not more fit than our nonathletes. Therefore, in Experiment 3, we will measure resting heart rate in order to confirm the physiological impact of the athletes’ regular exercise.”*

Minor point 13: 576 ’integrated fastest easily’ – improve wording

*Response: we have improved the wording to “In this context, congruent stimuli would be integrated more quickly and easily, resulting in a large Colavita effect…”*

Minor point 14: 588, Spence et al didn’t collect data did they, just a review of thother people’s observations that your findings contradict?

*Response: thank you for pointing this out, we have reworded the sentence to “Our findings include multiple observations that run contrary to Spence et al’s [21] conclusions that faster responses to visual information are associated with a greater number of ‘visual-only’ responses”*

Minor point 15: 596-597. Not sure anyone would disagree with last sentence of paper

*Response: We have rephrased the sentence as “Clearly, the speed at which visual stimuli are processed do not always predict the Colavita effect.”*

Minor point 16: Note Colavita himself published a couple subsequent studies on his namesake effect

*Response: this is certainly true, but we have focused on the studies that were the most relevant for our paper.*

Minor point 17: 685 note ref 33 . 58 repeated ….

*Response: thank you for pointing this out, the extra reference has been removed.*

Minor point 18: Why include Month in some of references?

*Response: we included month when it was available – some journals don’t list it.*

Reviewer #2

Minor point 1: Methods: Although the participant numbers look healthy, a power analysis would be useful to confirm this given the various different types of trial.

*Response: considering the Bayesian analysis, instead of an a-priori power analysis to set the sample size we used a specific criterion “We expected our manipulation to impact the pattern of errors in response to bimodal trials; we therefore used a criterion based on the overall number of bimodal errors to determine sample size. Data were collected until we had clear support for an effect of stimulus modality where there were more errors for bimodal trials than for unimodal trials.” (lines 147-151). Also, this is a within-subjects design – all participants are presented with all types of trials.*

Minot point 2: In Study 2 there are only 12 male participants, and I wonder if it would be useful to breakdown the gender differences in the athlete and non-athlete group as it’s not clear if there is a gender difference in responses or whether a majority of those male participants landed in one group or the other.

*Response: there were exactly 6 male athletes and 6 male non-athletes in Experiment 2. We have added this information in the text “The sample consisted of 46 non-athletes (six identified as male), and 55 athletes (six identified as male).” (lines 286-287). Likewise there were 14 male athletes and 14 male non-athletes in Experiment 3. We have also added this information in the text “Fifty were varsity or club athletes and 54 were non-athletes. Participants’ mean age was 19.15 years (SD = 1.61) and 73 identified as female, 28 identified as male (14 were athletes) and 3 identified as other.” (lines 436-438)*

Minor point 3: On the athlete grouping, I’m not sure that the use of athletes in this circumstance is justified enough. Reaction time in an online task seems far removed from any particular physical ability differences.

*Response: our task was performed in person (not online), and the faster reaction times are not associated with physical ability, but rather with long-term neurological and cognitive effects associated with regular exercise. We have explained this in the text “The frequent cardiovascular exercise that athletes engage in has been repeatedly associated with faster responses on cognitive tasks like the Stroop task [25], visuo-spatial processing [26], a flanker task [27], an oddball task [28] and importantly, visual detection [29, 30].” (lines 262-266*)

Minor point 4: I think some discussion of alternative explanations/issues should be included. For instance, in making stimuli using spoken word auditory stimuli, there is a disconnect from the visual. The moo of a cow could be coming from the picture of a cow, but the word ‘cow’ could not. Do the authors think that this potential disconnect (where essentially 2 stimuli are being presented rather than 1 stimuli in 2 modalities) influences their findings?

*Response: This is an interesting point – thank you to the reviewer - but is not supported by our findings. We have addressed is at the beginning of the discussion: “Using spoken words as auditory stimuli could have reduced the Colavita effect by reducing ‘unity’ – the sound “cat” cannot come from the picture of a cat the same way the sound “meow” can come from the picture of a cat. However, the type of auditory stimuli used (sounds vs. spoken words) generally did not impact performance. In Experiment 2 the Colavita effect was larger – not smaller – when words were used as auditory stimuli, and this did not interact with the congruence of the audiovisual stimuli. This observation is inconsistent with the idea that word auditory stimuli would be less well connected to pictures than animal sounds.” (lines 541-548)*

Minor point 5: Also some discussion on the fact that word/language comprehension is different from non-language comprehension, and as such requires different cognitive resources. Could this difference influence reaction times/errors?

*Response: considering the limited impact of using word stimuli, we believe this would be speculative and beyond the scope of our paper. Though using names as auditory stimuli led to slower RTs for these stimuli compared to bimodal trials in Experiment 1, this effect was not replicated in Experiment 2. Also, using names as auditory stimuli did not impact errors in the general error analysis. It doesn’t seem useful to discuss to discuss the cognitive resources required for language comprehension when the use of linguistic stimuli had a limited impact on performance.*

Minor point 6: Furthermore, were all participants native English speakers as this may add a further barrier in a resource/reaction time sense?

*Response: Our sample included individuals for whom English was not a first language. We included participants irrespective of first language for multiple reasons. Firstly, the Colavita is a task where participants report the modality of a stimulus – the meaning of the stimulus is typically irrelevant. Secondly, because language is irrelevant, participants’ first language is not something typically controlled for – so we included ESL participants to more easily compare our results to past studies. Finally, though it is possible that for ESL individuals the connection between pictures and words is weaker, we would expect it to reduce the size of the effect we are looking for. We would expect the disconnect to lead to less unity between the visual and auditory stimuli, and therefore to a smaller Colavita effect and to smaller differences between congruent and incongruent stimuli. WE have addressed this in the first participants section of our paper (lines 156-164): “All participants were studying at an English-speaking institution and therefore should have a level of understanding of the English language that allows them to function in University. We therefore did not exclude participants whose first language was not English, and our sample included individuals for whom English was a second language (ESL). In the Colavita task, participants report the modality of stimuli – an understanding of the words presented is not necessary. It is possible that for ESL individuals the association between an English word and a picture is weaker, but we would expect this to reduce the size of the Colavita effect – we therefore used a conservative approach and included all participants irrespective of first language.”*

Minor point 7: I think as well some discussion on potential future work and/or some discussion of the phenomenon of the reverse Colavita effect (in which children (some refs below) have been shown to have the opposite dominance, responding to the auditory over the visual), would be very interesting. Especially if the authors think that these results could inform those studies as well.

*Response: though a discussion of the reversed Colavita in children might be quite interesting, it falls outside the scope of our paper. We have however added a final paragraph about some future directions; “Though we have shown that semantic correspondence impacts the Colavita effect, we demonstrated this using specific stimuli and it will be important to replicate the effect using additional stimuli in order to make the findings more generalizable. It would also be useful to evaluate whether congruence impacts the auditory suppression observed by other researchers [34]. Importantly, we have shown that a Colavita effect can be obtained with verbal auditory stimuli (the names of objects), which will hopefully facilitate stimulus selection in future research.” (lines 621-626)*
