# Supplementary material for: Semantic congruence impacts audiovisual processing in the Colavita effect
Source: PLoS One. 2026 Jul 6;21(7):e0352876. doi: 10.1371/journal.pone.0352876 (PMC13336217; doi:10.1371/journal.pone.0352876)
Supplement: Attachment — Submitted filename: PLOS_REV_Responses.docx [file pone.0352876.s003.docx]

Responses to reviewer

Reviewer 1

Comment #1: Ref 10. was published 2016, not 2015

*Our response: thank you for pointing out this error, this reference has been updated (it is now reference #14).*

Comment #2: ref 3 and 36 are the same! Perhaps 1 should be replaced by Sinnett, S., Spence, C., & Soto-Faraco, S. (2007). Visual dominance and attention: The Colavita effect revisited. Perception & Psychophysics, 69, 673-686.

*Our response: Reference 36 was in fact meant to be reference 3. Reference 36 has been removed and numbers have been updated in the rest of the document.*

Comment #3: Many relevant papers not mentioned

eg audiotactile and visuotactile Colavita effect

Occelli, V., Hartcher O’Brien, J., Spence, C., & Zampini, M. (2010). Assessing the audiotactile Colavita effect in near and rear space. Experimental Brain Research, 203, 517-532.

Hartcher-O’Brien, J., Gallace, A., Krings, B., Koppen, C., & Spence, C. (2008). When vision ‘extinguishes’ touch in neurologically-normal people: Extending the Colavita visual dominance effect. Experimental Brain Research, 186, 643-658.

*Our response: thank you for the suggestion, these two articles have been added into the front end of the manuscript when the Colavita effect is introduced (lines 41-42). “The Colavita effect is a reliably demonstrated effect [3, 4, 5], persisting through manipulations of response characteristics including: number of keys used [2], number of targets requiring a response [6], proportion of trials in different modalities [2,6,7], stimulus intensity [1], the addition of a secondary task [8], and the type of stimulus being presented (e.g., audio-tactile, visuo-tactile) [9, 10].”*

And
Nava, E., & Pavani, F. (2013). Changes in sensory preference during childhood. Converging evidence from the Colavita effect and the sound-induced flash illusion. Child Development, 84, 604-616.

Hirst, R. J., Cragg, L., & Allen, H. A. (2018). Vision dominates in adults but not children: A meta-analysis of the Colavita effect. Neuroscience & Biobehavioural Reviews, 94, 286-301.

*Our response: The developmental trajectory of the Colavita effect is beyond the scope of our manuscript. We believe these references would not add to our manuscript and may in fact seem out of place.*

Colavita, F. B. (1982). Visual dominance and attention in space. Bulletin of the Psychonomic Society, 19, 261-262.
Colavita, F. B., Tomko, R., & Weisberg, D. (1976). Visual prepotency and eye orientation. Bulletin of the Psychonomic Society, 8, 25-26.
Colavita, F. B., & Weisberg, D. (1979). A further investigation of visual dominance. Perception & Psychophysics, 25, 345-347.

*Our response: thank you for the suggestion, these three articles have been added into the front end of the manuscript when the Colavita effect is introduced (lines 41-42). “The Colavita effect is a reliably demonstrated effect [3, 4, 5], persisting through manipulations of response characteristics including: number of keys used [2], number of targets requiring a response [6], proportion of trials in different modalities [2,6,7], stimulus intensity [1], the addition of a secondary task [8], and the type of stimulus being presented (e.g., audio-tactile, visuo-tactile) [9, 10].”*

Egeth, H. E., & Sager, L. C. (1977). On the locus of visual dominance. Perception & Psychophysics, 22, 77-86.

*Our response: This article was already included prior to revisions (reference #4 in our last submission, now reference #6).*

Comment #4: Figure 2 legend incomplete

*Our response: thank you for pointing this out, this has been corrected*

Comment #5: Figure 3 and 3 axes labels inconsistent

*Our response: all axes are now labeled as ‘Mean Error Rate’*

Comment #6: Note every figure legend needs to make clear which expt data refer to, and also what your error bars indicate.

*Our response: these have been updated*

Comment #7: Line 77 you completely misdescribe the study, which has nothing to do with synaesthetes!!

*Our response: thank you for pointing out this confusion. The text has been updated to better reflect the method and findings of the study, with a focus on the neural patterns observed despite no difference in behavioural patterns*.

Comment #8: Line 98 provide more details

Our response: the description of this specific article was expanded to include relevant details of the experiments: “They conducted a series of three search task experiments manipulating perceptual load and task relevance and found that relevant auditory stimuli generally accelerated search times but only in low-load conditions. The authors concluded that the automaticity of crossmodal semantic congruency may depend on attentional load.”

Comment #9: Sample size seems reasonablly large, but these days needmore formal power analysis to convince you have sufficient data to come to meaningful conclusions

*Our response: Bayesian analyses already provide information about the probability of obtaining a finding if the null hypothesis is true vs if the alternative hypothesis is true. However, at the end of Experiment 1 we added a power analysis that focused on our ability to detect the Colavita effect and used this information to confirm the sample sizes for Experiment 2 and Experiment 3. “To confirm our sample size and make sure we would have enough participants to detect the Colavita effect, we calculated the effect size for the Colavita effect observed in Experiment 1: partial η^2^ = .342 (power was near 1.00). Given the effect size we used the G-power software to confirm that a minimum sample size of 30 per group would be sufficient to detect the effect with 99% power, but aimed to recruit a minimum of 45 participants per group to compensate for the unknown effect size for semantic correspondence.”*

Comment #10: 156 why University uppercase?

*Our response: thank you for pointing this out, it has been updated to lower case.*

Comment #11: 165-170 can you confirm sound started at immediate onset of sound file?

*Our response it does. We have updated the description to mention this: “all auditory stimuli were presented for 350ms at 65dB through speakers at both sides of the computer screen, with the sounds starting immediately at the onset of the sound file”.*

Comment #12: 193 trials where the response was correct - do you mean?

*Our response: yes, trials where the response was correct – we have updated the text: “Reaction times (RT) to trials where the response was correct were trimmed recursively at 3 SDs…”*

Comment #13: I got a bit confused whether mean error rate or proportion of errors, either was isn't the magnitude of Colavita effect miniscule? .something difference. So in any meaningful sense, this effect is so tiny as to be meaningless?

*Our response: In the Colavita task, participants are asked to report the modality of stimuli (auditory, visual, or bimodal), which is a fairly simple task where error rates should be quite low. Indeed, error rates in response to unimodal trials are quite low (2-3% in our experiments, or error rates of .02 or .03). This is why it is so telling that error rates rise so much in response to bimodal trials (8 to 15% in our experiments, or error rates of .08 to .15). These observations are consistent with previous research and have been replicated on several occasions by other researchers. The error rates in response to bimodal trials are then split into the type of responses provided by participants: ‘visual-only’ or ‘auditory-only,’ which clearly reduces the magnitude of error rates reported, but the effect size associated with this difference is quite large (see comment above – our effect size in Experiment 1 was .342 for the Colavita effect). What is important is the asymmetrical pattern observed when these are split, where there are consistently (in adults) more ’visual-only’ than ‘auditory-only’ errors. Again, these observations are consistent with previous research and have been replicated on several occasions by other researchers. These consistent differences are meaningful and have spurred discussion about visual dominance, sensitivity to visual and auditory stimuli, sensory development, etc.*

Comment #14: Once you have introduced RT acronym stick with it.

*Our response: All subsequent reaction time statements were updated to RT.*

Comment #15: 432 non-athlete seems wrong, you mean people whop were not in athetic team, whether or not they were athletic?

*Our response: A statement has been added to clarify what athlete and non-athlete refers to: “In this series of studies, the term athlete referred to any participant who self-reported as being an athlete member of a sport team (individual or team sport), and the term non-athlete referred to all other participants.”*

Comment #16: 459. are 3 dp for bpm warranted?

*Our response: We are using 3 decimal places to be consistent with the rest of the paper*

Comment #17: 538-539. Sorry, to me these 2 copnditions the same, I never heard a meow coming out of a static pitcure? Have you?

*Our response: thank you for pointing this out. Generally, research on the audiovisual Colavita effect uses two-dimensional stimuli that cannot by themselves produce sounds. We have rephrased to avoid confusion: “This means that when presented with the sound “cat” and a picture of a cat, the auditory stimulus is not facilitating detection of the visual stimulus, as shown in another study of the Colavita effect [6].”*

Comment #18: 605-606 I presume the Spence et al conclusion refers to relatively faster RTs to vision (than audition) not the absolute speed of response which is likely irrelevant to sensory dominance?

*Our response: thank you for pointing this out. We have rephrased this section to clarify things: “Our findings include multiple observations that run contrary to evidence from another Colavita study (see Koppen & Spence [2]). In Koppen and Spence’s Experiment 3 [2] visual-only errors on bimodal trials were significantly faster than unimodal visual trials and correctly reported bimodal trials. While reviewing this finding, Spence et al. [29] conclude that ‘visual-only’ responses to bimodal trials are tightly linked to speed of response.”*
