# Supplementary material for: Semantic congruence impacts audiovisual processing in the Colavita effect
Source: PLoS One. 2026 Jul 6;21(7):e0352876. doi: 10.1371/journal.pone.0352876 (PMC13336217; doi:10.1371/journal.pone.0352876)
Supplement: Attachment — Submitted filename: Responses_to_reviewer.docx [file pone.0352876.s004.docx]

Responses to Academic Editor

The academic editor did not raise any specific point

Responses to reviewer

Point 1: Avoid starting sentence with also “145 the unity effect [11]. Also,…”

Our response: we have rephrased the sentence as “Chainey and Humphreys [28] have also shown that…”

Point 2: Avoid personal pronouns “383 Finally, we separated the errors”

Our response: Though the PLOS writing center does not specify a preference for first-person pronouns or the use of active/ passive writing voices, the use of first-person pronouns is permitted and encouraged in both APA and AMA writing styles to improve writing clarity.

Point 3: “474 A planned Bayesian independent samples t-test confirmed that the heart rate of athletes (M = 475 73.660 bpm) was lower than the heart rate of nonathletes (M = 87.389 bpm), BF10 = 2631.” Too many decimal places.

Our response: We have updated our reporting to reflect Lang and Altman’s (2015) SIMPL guidelines (which PLOS references for statistical reporting guidelines) who indicate to round to an "appropriate degree of precision." For bpm (measured with a pulse oximeter that provides whole numbers) this was reduced to one decimal place. Mean age was reported as years and months on all values, so two decimal points is appropriate in this case. Similar logic was maintained for error rate reporting (rounded to 2 decimal places). Reporting BF values is specified in the Open Science Framework as being reported to 2 decimal places for greater than 1, so all BF values were updated as such.

Point 4: Missing space “477 RTfor correct”

Our response: Thank you for pointing out this typo, it has been corrected.

Point 5:

1. Colavita FB. Human sensory dominance. Perception & Psychophysics. 1974 Mar;16(2):409–12. 644 doi:10.3758/BF03203962
645 2. Koppen C, Spence C. Seeing the light: Exploring the Colavita visual dominance effect. Exp Brain Res. 646 2007 Feb 27;180:737-54. doi:10.1007/s00221-007-0894-3
No need to include month of publication in references

Our response: Months were removed from each reference.

Point 6: Probably error on reference number formatting “Colavita FB. Visual dominance and attention in space. Bulletin of the 651 Psychonomic Society. 1982
652 May;19(5):261-2. doi:10.3758/BF03330251 Sinnett S, Soto-Faraco S, Spence C. The co-occurrence of
653 multisensory competition and facilitation. Acta Psychologica. 2008 May;128(1):153–61.
654 doi:10.1016/j.actpsy.2007.12.002”

Our response: Thank you for pointing this duplication, it has been corrected.

Point 7: References often incocnsistent in whether a space after :?

Our response: corrections were made to all relevant articles.
