# Supplementary material for: Semantic congruence impacts audiovisual processing in the Colavita effect
Source: PLoS One. 2026 Jul 6;21(7):e0352876. doi: 10.1371/journal.pone.0352876 (PMC13336217; doi:10.1371/journal.pone.0352876)
Supplement: Peer review history

# Peer review: Decision Letter 0

**DOI:** 10.1371/journal.pone.0352876.r001

**Authors:** Elisa Scerrati

28 Jan 2026

PONE-D-25-52734

Semantic congruence impacts audiovisual processing in the Colavita effect

PLOS One

Dear Dr. Desmarais,

Thank you for submitting your manuscript to PLOS ONE. After careful consideration, we feel that it has merit but does not fully meet PLOS ONE’s publication criteria as it currently stands. Therefore, we invite you to submit a revised version of the manuscript that addresses the points raised during the review process.

Your paper has now been reviewed by two experts in the field, and I am pleased to inform you that it has been recommended for publication pending minor revisions. Both reviewers have provided detailed line-by-line comments that should be addressed systematically. These include methodological clarifications, terminology refinements and additional references you may want to discuss.

Please submit your revised manuscript by Mar 14 2026 11:59PM. If you will need more time than this to complete your revisions, please reply to this message or contact the journal office at plosone@plos.org. When you're ready to submit your revision, log on to https://www.editorialmanager.com/pone/ and select the 'Submissions Needing Revision' folder to locate your manuscript file.

Please include the following items when submitting your revised manuscript:

- A letter that responds to each point raised by the academic editor and reviewer(s). You should upload this letter as a separate file labeled 'Response to Reviewers'.
- A marked-up copy of your manuscript that highlights changes made to the original version. You should upload this as a separate file labeled 'Revised Manuscript with Track Changes'.
- An unmarked version of your revised paper without tracked changes. You should upload this as a separate file labeled 'Manuscript'.

-->If you would like to make changes to your financial disclosure, please include your updated statement in your cover letter. Guidelines for resubmitting your figure files are available below the reviewer comments at the end of this letter.

If applicable, we recommend that you deposit your laboratory protocols in protocols.io to enhance the reproducibility of your results. Protocols.io assigns your protocol its own identifier (DOI) so that it can be cited independently in the future. For instructions see: https://journals.plos.org/plosone/s/submission-guidelines#loc-laboratory-protocols. Additionally, PLOS ONE offers an option for publishing peer-reviewed Lab Protocol articles, which describe protocols hosted on protocols.io. Read more information on sharing protocols at https://plos.org/protocols?utm_medium=editorial-email&utm_source=authorletters&utm_campaign=protocols.

We look forward to receiving your revised manuscript.

Kind regards,

Elisa Scerrati

Academic Editor

PLOS One

Journal Requirements:

When submitting your revision, we need you to address these additional requirements.

1. Please ensure that your manuscript meets PLOS ONE's style requirements, including those for file naming. The PLOS ONE style templates can be found at https://journals.plos.org/plosone/s/file?id=wjVg/PLOSOne_formatting_sample_main_body.pdf and https://journals.plos.org/plosone/s/file?id=ba62/PLOSOne_formatting_sample_title_authors_affiliations.pdf

2. Please include your full ethics statement in the ‘Methods’ section of your manuscript file. In your statement, please include the full name of the IRB or ethics committee who approved or waived your study, as well as whether or not you obtained informed written or verbal consent. If consent was waived for your study, please include this information in your statement as well.

3. When completing the data availability statement of the submission form, you indicated that you will make your data available on acceptance. We strongly recommend all authors decide on a data sharing plan before acceptance, as the process can be lengthy and hold up publication timelines. Please note that, though access restrictions are acceptable now, your entire data will need to be made freely accessible if your manuscript is accepted for publication. This policy applies to all data except where public deposition would breach compliance with the protocol approved by your research ethics board. If you are unable to adhere to our open data policy, please kindly revise your statement to explain your reasoning and we will seek the editor's input on an exemption. Please be assured that, once you have provided your new statement, the assessment of your exemption will not hold up the peer review process.

4. Thank you for stating the following financial disclosure:

This work was supported by a Natural Science and Engineering Research Council of Canada grant (RGPIN-2020-05049) awarded to the last author.

Please state what role the funders took in the study. If the funders had no role, please state: "The funders had no role in study design, data collection and analysis, decision to publish, or preparation of the manuscript."

If this statement is not correct you must amend it as needed.

Please include this amended Role of Funder statement in your cover letter; we will change the online submission form on your behalf.

5. Thank you for stating the following in the Acknowledgments Section of your manuscript:

This work was supported by a Natural Science and Engineering Research Council of Canada grant (RGPIN-2020-05049) awarded to the last author.

We note that you have provided funding information that is not currently declared in your Funding Statement. However, funding information should not appear in the Acknowledgments section or other areas of your manuscript. We will only publish funding information present in the Funding Statement section of the online submission form.

Please remove any funding-related text from the manuscript and let us know how you would like to update your Funding Statement. Currently, your Funding Statement reads as follows:

This work was supported by a Natural Science and Engineering Research Council of Canada grant (RGPIN-2020-05049) awarded to the last author.

Please include your amended statements within your cover letter; we will change the online submission form on your behalf.

6. If the reviewer comments include a recommendation to cite specific previously published works, please review and evaluate these publications to determine whether they are relevant and should be cited. There is no requirement to cite these works unless the editor has indicated otherwise.

7. Please review your reference list to ensure that it is complete and correct. If you have cited papers that have been retracted, please include the rationale for doing so in the manuscript text, or remove these references and replace them with relevant current references. Any changes to the reference list should be mentioned in the rebuttal letter that accompanies your revised manuscript. If you need to cite a retracted article, indicate the article’s retracted status in the References list and also include a citation and full reference for the retraction notice.

[Note: HTML markup is below. Please do not edit.]

Reviewers' comments:

Reviewer's Responses to Questions

**Comments to the Author**

1. Is the manuscript technically sound, and do the data support the conclusions?

The manuscript must describe a technically sound piece of scientific research with data that supports the conclusions. Experiments must have been conducted rigorously, with appropriate controls, replication, and sample sizes. The conclusions must be drawn appropriately based on the data presented.

Reviewer #1: Yes

Reviewer #2: Yes

**********

2. Has the statistical analysis been performed appropriately and rigorously?

Reviewer #1: I Don't Know

Reviewer #2: Yes

**********

3. Have the authors made all data underlying the findings in their manuscript fully available?

The PLOS Data policy requires authors to make all data underlying the findings described in their manuscript fully available without restriction, with rare exception (please refer to the Data Availability Statement in the manuscript PDF file). The data should be provided as part of the manuscript or its supporting information, or deposited to a public repository. For example, in addition to summary statistics, the data points behind means, medians and variance measures should be available. If there are restrictions on publicly sharing data—e.g. participant privacy or use of data from a third party—those must be specified.

Reviewer #1: No

Reviewer #2: No

**********

4. Is the manuscript presented in an intelligible fashion and written in standard English?

PLOS ONE does not copyedit accepted manuscripts, so the language in submitted articles must be clear, correct, and unambiguous. Any typographical or grammatical errors should be corrected at revision, so please note any specific errors here.

Reviewer #1: Yes

Reviewer #2: Yes

**********

5. Review Comments to the Author

Please use the space provided to explain your answers to the questions above. You may also include additional comments for the author, including concerns about dual publication, research ethics, or publication ethics. (Please upload your review as an attachment if it exceeds 20,000 characters)

Reviewer #1: This generally appears to be a sensibly conducted and appropriately analyzed series of studies on the Colavita visual dominance effect.

Mostly minor suggestions for improvement and clarification.

l.16 ‘Robust’ – not sure about that. Often-demonstrated yes, but a very small effect,

l. 18 ‘often’ – I would say ‘occasionally’

l. 39 ‘quite robust’ – backpeddling?

l. 68 provide reference on this.

l. 93 Note also Vatakis, A., Ghazanfar, A., & Spence, C. (2008). Facilitation of multisensory integration by the “unity effect” reveals that speech is special. Journal of Vision, 8/9/14, 1-11. https://doi.org/10.1167/8.9.14.

174. Given that p(bimodal) influences likelihood of observing the Colavita effect, is the % here quite high

194 |Avoid starting sentence with ‘Also…’

246 ‘could occur’ tense seems wrong

260’traditional Colavita task’ sounds too strong a claim

281-282 More details on classification helpful, and presumably an arbitraty cut-off, and that many athletes may not be part of club or team?

326 add ‘,’

391 Improve start of sentence

576 ’integrated fastest easily’ – improve wording

588, Spence et al didn’t collect data did they, just a review of thother people’s observations that your findings contradict?

596-597. Not sure anyone would disagree with last sentence of paper

Note Colavita himself published a couple subsequent studies on his namesake effect

685 note ref 33 . 58 repeated ….

Why include Month in some of references?

Reviewer #2: I think the study does a good job of explaining the background, and justifying the experiments.

I have some minor comments.

Methods: Although the participant numbers look healthy, a power analysis would be useful to confirm this given the various different types of trial.

In Study 2 there are only 12 male participants, and I wonder if it would be useful to breakdown the gender differences in the athlete and non-athlete group as it’s not clear if there is a gender difference in responses or whether a majority of those male participants landed in one group or the other.

On the athlete grouping, I’m not sure that the use of athletes in this circumstance is justified enough. Reaction time in an online task seems far removed from any particular physical ability differences.

Discussion:

I think some discussion of alternative explanations/issues should be included. For instance, in making stimuli using spoken word auditory stimuli, there is a disconnect from the visual. The moo of a cow could be coming from the picture of a cow, but the word ‘cow’ could not. Do the authors think that this potential disconnect (where essentially 2 stimuli are being presented rather than 1 stimuli in 2 modalities) influences their findings?

Also some discussion on the fact that word/language comprehension is different from non-language comprehension, and as such requires different cognitive resources. Could this difference influence reaction times/errors?

Furthermore, were all participants native English speakers as this may add a further barrier in a resource/reaction time sense?

I think as well some discussion on potential future work and/or some discussion of the phenomenon of the reverse Colavita effect (in which children (some refs below) have been shown to have the opposite dominance, responding to the auditory over the visual), would be very interesting. Especially if the authors think that these results could inform those studies as well.

Sloutsky and Napolitano (2003)

Nava and Pavani (2013)

Wille and Ebersbach (2016)

Ross, Atkins, Allison, Simpson, Duffell, Williams & Ermolina(2021)

Ross, Williams, Herbert, Manning & Lee (2023)

Navi and Pavani 12

Meta Analysis in Hirst, Cragg & Allen (2018)

**********

6. PLOS authors have the option to publish the peer review history of their article (what does this mean?). If published, this will include your full peer review and any attached files.

If you choose “no”, your identity will remain anonymous but your review may still be made public.

**Do you want your identity to be public for this peer review?**  For information about this choice, including consent withdrawal, please see our Privacy Policy.

Reviewer #1: No

Reviewer #2: No

**********

[NOTE: If reviewer comments were submitted as an attachment file, they will be attached to this email and accessible via the submission site. Please log into your account, locate the manuscript record, and check for the action link "View Attachments". If this link does not appear, there are no attachment files.]

To ensure your figures meet our technical requirements, please review our figure guidelines: https://journals.plos.org/plosone/s/figures

You may also use PLOS’s free figure tool, NAAS, to help you prepare publication quality figures: https://journals.plos.org/plosone/s/figures#loc-tools-for-figure-preparation.

NAAS will assess whether your figures meet our technical requirements by comparing each figure against our figure specifications.

# Author comment: Author response to Decision Letter 1

**DOI:** 10.1371/journal.pone.0352876.r002

17 Mar 2026

To the editor: we have made the following changes

Editor request #2 – the full ethics statement is included in the method section. It includes the full name of the ethics committee and the fact that we obtained written consent. It appears at the beginning of Experiment 1.

Editor request #3 – the data has been made available on OSF at the following link

https://osf.io/dzc6a/overview?view_only=dc5d18132c78488087bc01d67db2a473

Editor request #4/5 – we have removed the statement about funding from the acknowledgements. The full funding statement should be:

This work was supported by a Natural Science and Engineering Research Council of Canada grant (RGPIN-2020-05049) awarded to the last author. The funders had no role in study design, data collection and analysis, decision to publish, or preparation of the manuscript.

Editor request #6 – we have explained to the reviewer that the requested topic of discussion and additional references were beyond the scope of this article.

To the reviewers:

Reviewer #1

Minor point 1: l.16 ‘Robust’ – not sure about that. Often-demonstrated yes, but a very small effect,

Response: we have removed the term ‘robust’- this has been done throughout the manuscript for consistency.

Minor point 2: l. 18 ‘often’ – I would say ‘occasionally’

Response: we have replaced ‘often’ with ‘occasionally’

Minor point 3: l. 39 ‘quite robust’ – backpeddling?

Response: we have replaced the term ‘quite robust’ with ‘reliably demonstrated effect’

Minor point 4: l. 68 provide reference on this.

Response: the statement is a suggestion as to what might have happened, not a reference to specific previous findings. We have nonetheless referenced a relevant past finding: “This stability through repeated pairings can lead to an association in memory (see James and Gauthier 2003 for example and Murray and Shams 2023 for a review) – after a few pairings the two elements might bind more easily and give rise to a single perceptual event.”(lines 67-69)

Minor point 5: l. 93 Note also Vatakis, A., Ghazanfar, A., & Spence, C. (2008). Facilitation of multisensory integration by the “unity effect” reveals that speech is special. Journal of Vision, 8/9/14, 1-11. https://doi.org/10.1167/8.9.14.

Response: we have added the reference.

Minor point 6: 174. Given that p(bimodal) influences likelihood of observing the Colavita effect, is the % here quite high

Response: the proportion of auditory, visual, and bimodal trials (20% of bimodal trials) is consistent with our past studies to produce a Colavita effect. It is also consistent with other studies of the Colavita effect. Koppen and Spence replicated the effect using 40/40/20 and 33/33/33 and reported that: “These results therefore suggest that stimulus probability and the response demands of the task do not contribute significantly to the Colavita effect”

Minor point 7: 194 |Avoid starting sentence with ‘Also…’

Response: we have removed ‘Also’ from the sentence.

Minor point 8: 246 ‘could occur’ tense seems wrong

Response: we have replaced ‘could occur’ with ‘might have occurred.’

Minor point 9: 260’traditional Colavita task’ sounds too strong a claim

Response: we replaced ‘traditional Colavita task’ with ‘Colavita task containing pictures and sounds’

Minor point 10: 281-282 More details on classification helpful, and presumably an arbitraty cut-off, and that many athletes may not be part of club or team?

Response: We clarified that all individuals that reported being part of a sports team were classified as athletes: “Individuals who reported being part of a sports team (whether a club or their university’s varsity team) within the last year were considered athletes,it is therefore possible that athletic individuals were included in the non-athlete group.”

Minor point 11: 326 add ‘,’

Response: a comma has been added

Minor point 12: 391 Improve start of sentence

Response: we have rephrased the sentence “. It is possible that despite practicing a sport, our athletes were not more fit than our nonathletes. Therefore, in Experiment 3, we will measure resting heart rate in order to confirm the physiological impact of the athletes’ regular exercise.”

Minor point 13: 576 ’integrated fastest easily’ – improve wording

Response: we have improved the wording to “In this context, congruent stimuli would be integrated more quickly and easily, resulting in a large Colavita effect…”

Minor point 14: 588, Spence et al didn’t collect data did they, just a review of thother people’s observations that your findings contradict?

Response: thank you for pointing this out, we have reworded the sentence to “Our findings include multiple observations that run contrary to Spence et al’s [21] conclusions that faster responses to visual information are associated with a greater number of ‘visual-only’ responses”

Minor point 15: 596-597. Not sure anyone would disagree with last sentence of paper

Response: We have rephrased the sentence as “Clearly, the speed at which visual stimuli are processed do not always predict the Colavita effect.”

Minor point 16: Note Colavita himself published a couple subsequent studies on his namesake effect

Response: this is certainly true, but we have focused on the studies that were the most relevant for our paper.

Minor point 17: 685 note ref 33 . 58 repeated ….

Response: thank you for pointing this out, the extra reference has been removed.

Minor point 18: Why include Month in some of references?

Response: we included month when it was available – some journals don’t list it.

Reviewer #2

Minor point 1: Methods: Although the participant numbers look healthy, a power analysis would be useful to confirm this given the various different types of trial.

Response: considering the Bayesian analysis, instead of an a-priori power analysis to set the sample size we used a specific criterion “We expected our manipulation to impact the pattern of errors in response to bimodal trials; we therefore used a criterion based on the overall number of bimodal errors to determine sample size. Data were collected until we had clear support for an effect of stimulus modality where there were more errors for bimodal trials than for unimodal trials.” (lines 147-151). Also, this is a within-subjects design – all participants are presented with all types of trials.

Minot point 2: In Study 2 there are only 12 male participants, and I wonder if it would be useful to breakdown the gender differences in the athlete and non-athlete group as it’s not clear if there is a gender difference in responses or whether a majority of those male participants landed in one group or the other.

Response: there were exactly 6 male athletes and 6 male non-athletes in Experiment 2. We have added this information in the text “The sample consisted of 46 non-athletes (six identified as male), and 55 athletes (six identified as male).” (lines 286-287). Likewise there were 14 male athletes and 14 male non-athletes in Experiment 3. We have also added this information in the text “Fifty were varsity or club athletes and 54 were non-athletes. Participants’ mean age was 19.15 years (SD = 1.61) and 73 identified as female, 28 identified as male (14 were athletes) and 3 identified as other.” (lines 436-438)

Minor point 3: On the athlete grouping, I’m not sure that the use of athletes in this circumstance is justified enough. Reaction time in an online task seems far removed from any particular physical ability differences.

Response: our task was performed in person (not online), and the faster reaction times are not associated with physical ability, but rather with long-term neurological and cognitive effects associated with regular exercise. We have explained this in the text “The frequent cardiovascular exercise that athletes engage in has been repeatedly associated with faster responses on cognitive tasks like the Stroop task [25], visuo-spatial processing [26], a flanker task [27], an oddball task [28] and importantly, visual detection [29, 30].” (lines 262-266)

Minor point 4: I think some discussion of alternative explanations/issues should be included. For instance, in making stimuli using spoken word auditory stimuli, there is a disconnect from the visual. The moo of a cow could be coming from the picture of a cow, but the word ‘cow’ could not. Do the authors think that this potential disconnect (where essentially 2 stimuli are being presented rather than 1 stimuli in 2 modalities) influences their findings?

Response: This is an interesting point – thank you to the reviewer - but is not supported by our findings. We have addressed is at the beginning of the discussion: “Using spoken words as auditory stimuli could have reduced the Colavita effect by reducing ‘unity’ – the sound “cat” cannot come from the picture of a cat the same way the sound “meow” can come from the picture of a cat. However, the type of auditory stimuli used (sounds vs. spoken words) generally did not impact performance. In Experiment 2 the Colavita effect was larger – not smaller – when words were used as auditory stimuli, and this did not interact with the congruence of the audiovisual stimuli. This observation is inconsistent with the idea that word auditory stimuli would be less well connected to pictures than animal sounds.” (lines 541-548)

Minor point 5: Also some discussion on the fact that word/language comprehension is different from non-language comprehension, and as such requires different cognitive resources. Could this difference influence reaction times/errors?

Response: considering the limited impact of using word stimuli, we believe this would be speculative and beyond the scope of our paper. Though using names as auditory stimuli led to slower RTs for these stimuli compared to bimodal trials in Experiment 1, this effect was not replicated in Experiment 2. Also, using names as auditory stimuli did not impact errors in the general error analysis. It doesn’t seem useful to discuss to discuss the cognitive resources required for language comprehension when the use of linguistic stimuli had a limited impact on performance.

Minor point 6: Furthermore, were all participants native English speakers as this may add a further barrier in a resource/reaction time sense?

Response: Our sample included individuals for whom English was not a first language. We included participants irrespective of first language for multiple reasons. Firstly, the Colavita is a task where participants report the modality of a stimulus – the meaning of the stimulus is typically irrelevant. Secondly, because language is irrelevant, participants’ first language is not something typically controlled for – so we included ESL participants to more easily compare our results to past studies. Finally, though it is possible that for ESL individuals the connection between pictures and words is weaker, we would expect it to reduce the size of the effect we are looking for. We would expect the disconnect to lead to less unity between the visual and auditory stimuli, and therefore to a smaller Colavita effect and to smaller differences between congruent and incongruent stimuli. WE have addressed this in the first participants section of our paper (lines 156-164): “All participants were studying at an English-speaking institution and therefore should have a level of understanding of the English language that allows them to function in University. We therefore did not exclude participants whose first language was not English, and our sample included individuals for whom English was a second language (ESL). In the Colavita task, participants report the modality of stimuli – an understanding of the words presented is not necessary. It is possible that for ESL individuals the association between an English word and a picture is weaker, but we would expect this to reduce the size of the Colavita effect – we therefore used a conservative approach and included all participants irrespective of first language.”

Minor point 7: I think as well some discussion on potential future work and/or some discussion of the phenomenon of the reverse Colavita effect (in which children (some refs below) have been shown to have the opposite dominance, responding to the auditory over the visual), would be very interesting. Especially if the authors think that these results could inform those studies as well.

Response: though a discussion of the reversed Colavita in children might be quite interesting, it falls outside the scope of our paper. We have however added a final paragraph about some future directions; “Though we have shown that semantic correspondence impacts the Colavita effect, we demonstrated this using specific stimuli and it will be important to replicate the effect using additional stimuli in order to make the findings more generalizable. It would also be useful to evaluate whether congruence impacts the auditory suppression observed by other researchers [34]. Importantly, we have shown that a Colavita effect can be obtained with verbal auditory stimuli (the names of objects), which will hopefully facilitate stimulus selection in future research.” (lines 621-626)

Attachment Submitted filename: Responses to reviewers.docx

# Peer review: Decision Letter 1

**DOI:** 10.1371/journal.pone.0352876.r003

**Authors:** Elisa Scerrati

13 Apr 2026

PONE-D-25-52734R1

Semantic congruence impacts audiovisual processing in the Colavita effect

PLOS One

Dear Dr. Desmarais,

Thank you for submitting your manuscript to PLOS ONE. After careful consideration, we feel that it has merit but does not fully meet PLOS ONE’s publication criteria as it currently stands. Therefore, we invite you to submit a revised version of the manuscript that addresses the points raised during the review process.

Following re-evaluation, Reviewer 2 accepted the manuscript in its current form, whereas Reviewer 1 has raised a number of concerns that necessitate further revisions. Requests concern revisions addressing the theoretical framing of the work and the accuracy of methodological reporting. Details are provided in the attached review.

Please submit your revised manuscript by May 28 2026 11:59PM. If you will need more time than this to complete your revisions, please reply to this message or contact the journal office at plosone@plos.org. When you're ready to submit your revision, log on to https://www.editorialmanager.com/pone/ and select the 'Submissions Needing Revision' folder to locate your manuscript file.

Please include the following items when submitting your revised manuscript:

- A letter that responds to each point raised by the academic editor and reviewer(s). You should upload this letter as a separate file labeled 'Response to Reviewers'.
- A marked-up copy of your manuscript that highlights changes made to the original version. You should upload this as a separate file labeled 'Revised Manuscript with Track Changes'.
- An unmarked version of your revised paper without tracked changes. You should upload this as a separate file labeled 'Manuscript'.

If you would like to make changes to your financial disclosure, please include your updated statement in your cover letter. Guidelines for resubmitting your figure files are available below the reviewer comments at the end of this letter.

If applicable, we recommend that you deposit your laboratory protocols in protocols.io to enhance the reproducibility of your results. Protocols.io assigns your protocol its own identifier (DOI) so that it can be cited independently in the future. For instructions see: https://journals.plos.org/plosone/s/submission-guidelines#loc-laboratory-protocols. Additionally, PLOS ONE offers an option for publishing peer-reviewed Lab Protocol articles, which describe protocols hosted on protocols.io. Read more information on sharing protocols at https://plos.org/protocols?utm_medium=editorial-email&utm_source=authorletters&utm_campaign=protocols.

As the corresponding author, your ORCID iD is verified in the submission system and will appear in the published article. PLOS supports the use of ORCID, and we encourage all coauthors to register for an ORCID iD and use it as well. Please encourage your coauthors to verify their ORCID iD within the submission system before final acceptance, as unverified ORCID iDs will not appear in the published article. *Only*  the individual author can complete the verification step; PLOS staff *cannot*  verify ORCID iDs on behalf of authors.

We look forward to receiving your revised manuscript.

Kind regards,

Elisa Scerrati

Academic Editor

PLOS One

Journal Requirements:

If the reviewer comments include a recommendation to cite specific previously published works, please review and evaluate these publications to determine whether they are relevant and should be cited. There is no requirement to cite these works unless the editor has indicated otherwise.

[Note: HTML markup is below. Please do not edit.]

Reviewers' comments:

Reviewer's Responses to Questions

**Comments to the Author**

1. If the authors have adequately addressed your comments raised in a previous round of review and you feel that this manuscript is now acceptable for publication, you may indicate that here to bypass the “Comments to the Author” section, enter your conflict of interest statement in the “Confidential to Editor” section, and submit your "Accept" recommendation.

Reviewer #1: (No Response)

Reviewer #2: All comments have been addressed

**********

2. Is the manuscript technically sound, and do the data support the conclusions?

The manuscript must describe a technically sound piece of scientific research with data that supports the conclusions. Experiments must have been conducted rigorously, with appropriate controls, replication, and sample sizes. The conclusions must be drawn appropriately based on the data presented.

Reviewer #1: (No Response)

Reviewer #2: Yes

**********

3. Has the statistical analysis been performed appropriately and rigorously?

Reviewer #1: (No Response)

Reviewer #2: Yes

**********

4. Have the authors made all data underlying the findings in their manuscript fully available?

The PLOS Data policy requires authors to make all data underlying the findings described in their manuscript fully available without restriction, with rare exception (please refer to the Data Availability Statement in the manuscript PDF file). The data should be provided as part of the manuscript or its supporting information, or deposited to a public repository. For example, in addition to summary statistics, the data points behind means, medians and variance measures should be available. If there are restrictions on publicly sharing data—e.g. participant privacy or use of data from a third party—those must be specified.

Reviewer #1: (No Response)

Reviewer #2: Yes

**********

5. Is the manuscript presented in an intelligible fashion and written in standard English?

PLOS ONE does not copyedit accepted manuscripts, so the language in submitted articles must be clear, correct, and unambiguous. Any typographical or grammatical errors should be corrected at revision, so please note any specific errors here.

Reviewer #1: Yes

Reviewer #2: Yes

**********

6. Review Comments to the Author

Please use the space provided to explain your answers to the questions above. You may also include additional comments for the author, including concerns about dual publication, research ethics, or publication ethics. (Please upload your review as an attachment if it exceeds 20,000 characters)

Reviewer #1: There are still many minorish problems with this ms.

Ref 10. was published 2016, not 2015

ref 3 and 36 are the same! Perhaps 1 should be replaced by Sinnett, S., Spence, C., & Soto-Faraco, S. (2007). Visual dominance and attention: The Colavita effect revisited. Perception & Psychophysics, 69, 673-686.

Many relevant papers not mentioned

eg audiotactile and visuotactile Colavita effect

Occelli, V., Hartcher O’Brien, J., Spence, C., & Zampini, M. (2010). Assessing the audiotactile Colavita effect in near and rear space. Experimental Brain Research, 203, 517-532.

Hartcher-O’Brien, J., Gallace, A., Krings, B., Koppen, C., & Spence, C. (2008). When vision ‘extinguishes’ touch in neurologically-normal people: Extending the Colavita visual dominance effect. Experimental Brain Research, 186, 643-658.

And

Nava, E., & Pavani, F. (2013). Changes in sensory preference during childhood. Converging evidence from the Colavita effect and the sound-induced flash illusion. Child Development, 84, 604-616.

Hirst, R. J., Cragg, L., & Allen, H. A. (2018). Vision dominates in adults but not children: A meta-analysis of the Colavita effect. Neuroscience & Biobehavioural Reviews, 94, 286-301.

Colavita, F. B. (1982). Visual dominance and attention in space. Bulletin of the Psychonomic Society, 19, 261-262.

Colavita, F. B., Tomko, R., & Weisberg, D. (1976). Visual prepotency and eye orientation. Bulletin of the Psychonomic Society, 8, 25-26.

Colavita, F. B., & Weisberg, D. (1979). A further investigation of visual dominance. Perception & Psychophysics, 25, 345-347.

Egeth, H. E., & Sager, L. C. (1977). On the locus of visual dominance. Perception & Psychophysics, 22, 77-86.

Figure 2 legend incomplete

Figure 3 and 3 axes labels inconsistent

Note every figure legend needs to make clear which expt data refer to, and also what your error bars indicate.

Line 77 you completely misdescribe the study, which has nothing to do with synaesthetes!!

Line 98 providemoredetails

Sample size seems reasonablly large, but these days needmore formal power analysis to convince you have sufficient data to come to meaningful conclusions

156 why University uppercase?

165-170 can you confirm sound started at immediate onset of sound file?

193 trials where the response was correct - do you mean?

I got a bit confused whether mean error rate or proportion of errors, either was isn't the magnitude of Colavita effect miniscule? .something difference. So in any meaningful sense, this effect is so tiny as to be meaningless?

Once you have introduced RT acronym stick with it.

432 non-athlete seems wrong, you mean people whop were not in athetic team, whether or not they were athletic?

459. are 3 dp for bpm warranted?

538-539. Sorry, to me these 2 copnditions the same, I never heard a meow coming out of a static pitcure? Have you?

605-606 I presume the Spence et al conclusion refers to relatively faster RTs to vision (than audition) not the absolute speed of response which is likely irrelevant to sensory dominance?

Reviewer #2: (No Response)

**********

7. PLOS authors have the option to publish the peer review history of their article (what does this mean?). If published, this will include your full peer review and any attached files.

If you choose “no”, your identity will remain anonymous but your review may still be made public.

**Do you want your identity to be public for this peer review?**  For information about this choice, including consent withdrawal, please see our Privacy Policy.

Reviewer #1: No

Reviewer #2: No

**********

[NOTE: If reviewer comments were submitted as an attachment file, they will be attached to this email and accessible via the submission site. Please log into your account, locate the manuscript record, and check for the action link "View Attachments". If this link does not appear, there are no attachment files.]

To ensure your figures meet our technical requirements, please review our figure guidelines: https://journals.plos.org/plosone/s/figures

You may also use PLOS’s free figure tool, NAAS, to help you prepare publication quality figures: https://journals.plos.org/plosone/s/figures#loc-tools-for-figure-preparation.

NAAS will assess whether your figures meet our technical requirements by comparing each figure against our figure specifications.

# Author comment: Author response to Decision Letter 2

**DOI:** 10.1371/journal.pone.0352876.r004

28 May 2026

To the editor:

Theoretical framing: the editor mentioned that the reviewer had concerned about theoretical framing of the work – we assumed this referred to the reviewer’s comment about papers that had not been cited. Though these papers were not central to the background of our work, we have nonetheless included most of them in the introduction where they could be incorporated. However, as per the journal requirements about suggestions to cite specific published works, we did not include the works on developmental aspects of the Colavita effect (Nava & Pavani 2013, Hirst, Cragg, & Allen, 2018) as they are not relevant to the current work.

Responses to reviewer

Reviewer 1

Comment #1: Ref 10. was published 2016, not 2015

Our response: thank you for pointing out this error, this reference has been updated (it is now reference #14).

Comment #2: ref 3 and 36 are the same! Perhaps 1 should be replaced by Sinnett, S., Spence, C., & Soto-Faraco, S. (2007). Visual dominance and attention: The Colavita effect revisited. Perception & Psychophysics, 69, 673-686.

Our response: Reference 36 was in fact meant to be reference 3. Reference 36 has been removed and numbers have been updated in the rest of the document.

Comment #3: Many relevant papers not mentioned

eg audiotactile and visuotactile Colavita effect

Occelli, V., Hartcher O’Brien, J., Spence, C., & Zampini, M. (2010). Assessing the audiotactile Colavita effect in near and rear space. Experimental Brain Research, 203, 517-532.

Hartcher-O’Brien, J., Gallace, A., Krings, B., Koppen, C., & Spence, C. (2008). When vision ‘extinguishes’ touch in neurologically-normal people: Extending the Colavita visual dominance effect. Experimental Brain Research, 186, 643-658.

Our response: thank you for the suggestion, these two articles have been added into the front end of the manuscript when the Colavita effect is introduced (lines 41-42). “The Colavita effect is a reliably demonstrated effect [3, 4, 5], persisting through manipulations of response characteristics including: number of keys used [2], number of targets requiring a response [6], proportion of trials in different modalities [2,6,7], stimulus intensity [1], the addition of a secondary task [8], and the type of stimulus being presented (e.g., audio-tactile, visuo-tactile) [9, 10].”

And

Nava, E., & Pavani, F. (2013). Changes in sensory preference during childhood. Converging evidence from the Colavita effect and the sound-induced flash illusion. Child Development, 84, 604-616.

Hirst, R. J., Cragg, L., & Allen, H. A. (2018). Vision dominates in adults but not children: A meta-analysis of the Colavita effect. Neuroscience & Biobehavioural Reviews, 94, 286-301.

Our response: The developmental trajectory of the Colavita effect is beyond the scope of our manuscript. We believe these references would not add to our manuscript and may in fact seem out of place.

Colavita, F. B. (1982). Visual dominance and attention in space. Bulletin of the Psychonomic Society, 19, 261-262.

Colavita, F. B., Tomko, R., & Weisberg, D. (1976). Visual prepotency and eye orientation. Bulletin of the Psychonomic Society, 8, 25-26.

Colavita, F. B., & Weisberg, D. (1979). A further investigation of visual dominance. Perception & Psychophysics, 25, 345-347.

Our response: thank you for the suggestion, these three articles have been added into the front end of the manuscript when the Colavita effect is introduced (lines 41-42). “The Colavita effect is a reliably demonstrated effect [3, 4, 5], persisting through manipulations of response characteristics including: number of keys used [2], number of targets requiring a response [6], proportion of trials in different modalities [2,6,7], stimulus intensity [1], the addition of a secondary task [8], and the type of stimulus being presented (e.g., audio-tactile, visuo-tactile) [9, 10].”

Egeth, H. E., & Sager, L. C. (1977). On the locus of visual dominance. Perception & Psychophysics, 22, 77-86.

Our response: This article was already included prior to revisions (reference #4 in our last submission, now reference #6).

Comment #4: Figure 2 legend incomplete

Our response: thank you for pointing this out, this has been corrected

Comment #5: Figure 3 and 3 axes labels inconsistent

Our response: all axes are now labeled as ‘Mean Error Rate’

Comment #6: Note every figure legend needs to make clear which expt data refer to, and also what your error bars indicate.

Our response: these have been updated

Comment #7: Line 77 you completely misdescribe the study, which has nothing to do with synaesthetes!!

Our response: thank you for pointing out this confusion. The text has been updated to better reflect the method and findings of the study, with a focus on the neural patterns observed despite no difference in behavioural patterns.

Comment #8: Line 98 provide more details

Our response: the description of this specific article was expanded to include relevant details of the experiments: “They conducted a series of three search task experiments manipulating perceptual load and task relevance and found that relevant auditory stimuli generally accelerated search times but only in low-load conditions. The authors concluded that the automaticity of crossmodal semantic congruency may depend on attentional load.”

Comment #9: Sample size seems reasonablly large, but these days needmore formal power analysis to convince you have sufficient data to come to meaningful conclusions

Our response: Bayesian analyses already provide information about the probability of obtaining a finding if the null hypothesis is true vs if the alternative hypothesis is true. However, at the end of Experiment 1 we added a power analysis that focused on our ability to detect the Colavita effect and used this information to confirm the sample sizes for Experiment 2 and Experiment 3. “To confirm our sample size and make sure we would have enough participants to detect the Colavita effect, we calculated the effect size for the Colavita effect observed in Experiment 1: partial η2 = .342 (power was near 1.00). Given the effect size we used the G-power software to confirm that a minimum sample size of 30 per group would be sufficient to detect the effect with 99% power, but aimed to recruit a minimum of 45 participants per group to compensate for the unknown effect size for semantic correspondence.”

Comment #10: 156 why University uppercase?

Our response: thank you for pointing this out, it has been updated to lower case.

Comment #11: 165-170 can you confirm sound started at immediate onset of sound file?

Our response it does. We have updated the description to mention this: “all auditory stimuli were presented for 350ms at 65dB through speakers at both sides of the computer screen, with the sounds starting immediately at the onset of the sound file”.

Comment #12: 193 trials where the response was correct - do you mean?

Our response: yes, trials where the response was correct – we have updated the text: “Reaction times (RT) to trials where the response was correct were trimmed recursively at 3 SDs…”

Comment #13: I got a bit confused whether mean error rate or proportion of errors, either was isn't the magnitude of Colavita effect miniscule? .something difference. So in any meaningful sense, this effect is so tiny as to be meaningless?

Our response: In the Colavita task, participants are asked to report the modality of stimuli (auditory, visual, or bimodal), which is a fairly simple task where error rates should be quite low. Indeed, error rates in response to unimodal trials are quite low (2-3% in our experiments, or error rates of .02 or .03). This is why it is so telling that error rates rise so much in response to bimodal trials (8 to 15% in our experiments, or error rates of .08 to .15). These observations are consistent with previous research and have been replicated on several occasions by other researchers. The error rates in response to bimodal trials are then split into the type of responses provided by participants: ‘visual-only’ or ‘auditory-only,’ which clearly reduces the magnitude of error rates reported, but the effect size associated with this difference is quite large (see comment above – our effect size in Experiment 1 was .342 for the Colavita effect). What is important is the asymmetrical pattern observed when these are split, where there are consistently (in adults) more ’visual-only’ than ‘auditory-only’ errors. Again, these observations are consistent with previous research and have been replicated on several occasions by other researchers. These consistent differences are meaningful and have spurred discussion about visual dominance, sensitivity to visual and auditory stimuli, sensory development, etc.

Comment #14: Once you have introduced RT acronym stick with it.

Our response: All subsequent reaction time statements were updated to RT.

Comment #15: 432 non-athlete seems wrong, you mean people whop were not in athetic team, whether or not they were athletic?

Our response: A statement has been added to clarify what athlete and non-athlete refers to: “In this series of studies, the term athlete referred to any participant who self-reported as being an athlete member of a sport team (individual or team sport), and the term non-athlete referred to all other participants.”

Comment #16: 459. are 3 dp for bpm warranted?

Our response: We are using 3 decimal places to be consistent with the rest of the paper

Comment #17: 538-539. Sorry, to me these 2 copnditions the same, I never heard a meow coming out of a static pitcure? Have you?

Our response: thank you for pointing this out. Generally, research on the audiovisual Colavita effect uses two-dimensional stimuli that cannot by themselves produce sounds. We have rephrased to avoid confusion: “This means that when presented with the sound “cat” and a picture of a cat, the auditory stimulus is not facilitating detection of the visual stimulus, as shown in another study of the Colavita effect [6].”

Comment #18: 605-606 I presume the Spence et al conclusion refers to relatively faster RTs to vision (than audition) not the absolute speed of response which is likely irrelevant to sensory dominance?

Our response: thank you for pointing this out. We have rephrased this section to clarify things: “Our findings include multiple observations that run contrary to evidence from another Colavita study (see Koppen & Spence [2]). In Koppen and Spence’s Experiment 3 [2] visual-only errors on bimodal trials were significantly faster than unimodal visual trials and correctly reported bimodal trials. While reviewing this finding, Spence et al. [29] conclude that ‘visual-only’ responses to bimodal trials are tightly linked to speed of response.”

Attachment Submitted filename: PLOS_REV_Responses.docx

# Peer review: Decision Letter 2

**DOI:** 10.1371/journal.pone.0352876.r005

**Authors:** Elisa Scerrati

11 Jun 2026

PONE-D-25-52734R2

Semantic congruence impacts audiovisual processing in the Colavita effect

PLOS One

Dear Dr. Desmarais,

Thank you for submitting your manuscript to PLOS ONE. After careful consideration, we feel that it has merit but does not fully meet PLOS ONE’s publication criteria as it currently stands. Therefore, we invite you to submit a revised version of the manuscript that addresses the points raised during the review process.

==============================

I thank the reviewer for their careful assessment. As only minor final corrections remain (largely stylistic and formatting issues), I invite the authors to submit a final revised version addressing these points, after which the manuscript can be accepted.

==============================

Please submit your revised manuscript by Jul 26 2026 11:59PM. If you will need more time than this to complete your revisions, please reply to this message or contact the journal office at plosone@plos.org. When you're ready to submit your revision, log on to https://www.editorialmanager.com/pone/ and select the 'Submissions Needing Revision' folder to locate your manuscript file.

Please include the following items when submitting your revised manuscript:

- A letter that responds to each point raised by the academic editor and reviewer(s). You should upload this letter as a separate file labeled 'Response to Reviewers'.
- A marked-up copy of your manuscript that highlights changes made to the original version. You should upload this as a separate file labeled 'Revised Manuscript with Track Changes'.
- An unmarked version of your revised paper without tracked changes. You should upload this as a separate file labeled 'Manuscript'.

If you would like to make changes to your financial disclosure, please include your updated statement in your cover letter. Guidelines for resubmitting your figure files are available below the reviewer comments at the end of this letter.

If applicable, we recommend that you deposit your laboratory protocols in protocols.io to enhance the reproducibility of your results. Protocols.io assigns your protocol its own identifier (DOI) so that it can be cited independently in the future. For instructions see: https://journals.plos.org/plosone/s/submission-guidelines#loc-laboratory-protocols. Additionally, PLOS ONE offers an option for publishing peer-reviewed Lab Protocol articles, which describe protocols hosted on protocols.io. Read more information on sharing protocols at https://plos.org/protocols?utm_medium=editorial-email&utm_source=authorletters&utm_campaign=protocols.

As the corresponding author, your ORCID iD is verified in the submission system and will appear in the published article. PLOS supports the use of ORCID, and we encourage all coauthors to register for an ORCID iD and use it as well. Please encourage your coauthors to verify their ORCID iD within the submission system before final acceptance, as unverified ORCID iDs will not appear in the published article. *Only*  the individual author can complete the verification step; PLOS staff *cannot*  verify ORCID iDs on behalf of authors.

We look forward to receiving your revised manuscript.

Kind regards,

Elisa Scerrati

Academic Editor

PLOS One

Journal Requirements:

If the reviewer comments include a recommendation to cite specific previously published works, please review and evaluate these publications to determine whether they are relevant and should be cited. There is no requirement to cite these works unless the editor has indicated otherwise.

Please review your reference list to ensure that it is complete and correct. If you have cited papers that have been retracted, please include the rationale for doing so in the manuscript text, or remove these references and replace them with relevant current references. Any changes to the reference list should be mentioned in the rebuttal letter that accompanies your revised manuscript. If you need to cite a retracted article, indicate the article’s retracted status in the References list and also include a citation and full reference for the retraction notice.

[Note: HTML markup is below. Please do not edit.]

Reviewers' comments:

Reviewer's Responses to Questions

**Comments to the Author**

1. If the authors have adequately addressed your comments raised in a previous round of review and you feel that this manuscript is now acceptable for publication, you may indicate that here to bypass the “Comments to the Author” section, enter your conflict of interest statement in the “Confidential to Editor” section, and submit your "Accept" recommendation.

Reviewer #1: (No Response)

**********

2. Is the manuscript technically sound, and do the data support the conclusions?

The manuscript must describe a technically sound piece of scientific research with data that supports the conclusions. Experiments must have been conducted rigorously, with appropriate controls, replication, and sample sizes. The conclusions must be drawn appropriately based on the data presented.

Reviewer #1: Yes

**********

3. Has the statistical analysis been performed appropriately and rigorously?

Reviewer #1: I Don't Know

**********

4. Have the authors made all data underlying the findings in their manuscript fully available?

The PLOS Data policy requires authors to make all data underlying the findings described in their manuscript fully available without restriction, with rare exception (please refer to the Data Availability Statement in the manuscript PDF file). The data should be provided as part of the manuscript or its supporting information, or deposited to a public repository. For example, in addition to summary statistics, the data points behind means, medians and variance measures should be available. If there are restrictions on publicly sharing data—e.g. participant privacy or use of data from a third party—those must be specified.

Reviewer #1: Yes

**********

5. Is the manuscript presented in an intelligible fashion and written in standard English?

PLOS ONE does not copyedit accepted manuscripts, so the language in submitted articles must be clear, correct, and unambiguous. Any typographical or grammatical errors should be corrected at revision, so please note any specific errors here.

Reviewer #1: Yes

**********

6. Review Comments to the Author

Please use the space provided to explain your answers to the questions above. You may also include additional comments for the author, including concerns about dual publication, research ethics, or publication ethics. (Please upload your review as an attachment if it exceeds 20,000 characters)

Reviewer #1: Improved in revision, though a number of minor final corrections.

Avoid starting sentence with also “145 the unity effect [11]. Also,…”

Avoid personal pronouns “383 Finally, we separated the errors”

“474 A planned Bayesian independent samples t-test confirmed that the heart rate of athletes (M =

475 73.660 bpm) was lower than the heart rate of nonathletes (M = 87.389 bpm), BF10 = 2631.” Too many decimal places.

Missing space “477 RTfor correct”

1. Colavita FB. Human sensory dominance. Perception & Psychophysics. 1974 Mar;16(2):409–12.

644 doi:10.3758/BF03203962

645 2. Koppen C, Spence C. Seeing the light: Exploring the Colavita visual dominance effect. Exp Brain Res.

646 2007 Feb 27;180:737-54. doi:10.1007/s00221-007-0894-3

No need to include month of publication in references

Probably error on reference number formatting “Colavita FB. Visual dominance and attention in space. Bulletin of the 651 Psychonomic Society. 1982

652 May;19(5):261-2. doi:10.3758/BF03330251 Sinnett S, Soto-Faraco S, Spence C. The co-occurrence of

653 multisensory competition and facilitation. Acta Psychologica. 2008 May;128(1):153–61.

654 doi:10.1016/j.actpsy.2007.12.002”

References often incocnsistent in whether a space after :?

**********

7. PLOS authors have the option to publish the peer review history of their article (what does this mean?). If published, this will include your full peer review and any attached files.

If you choose “no”, your identity will remain anonymous but your review may still be made public.

**Do you want your identity to be public for this peer review?**  For information about this choice, including consent withdrawal, please see our Privacy Policy.

Reviewer #1: No

**********

[NOTE: If reviewer comments were submitted as an attachment file, they will be attached to this email and accessible via the submission site. Please log into your account, locate the manuscript record, and check for the action link "View Attachments". If this link does not appear, there are no attachment files.]

To ensure your figures meet our technical requirements, please review our figure guidelines: https://journals.plos.org/plosone/s/figures

You may also use PLOS’s free figure tool, NAAS, to help you prepare publication quality figures: https://journals.plos.org/plosone/s/figures#loc-tools-for-figure-preparation.

NAAS will assess whether your figures meet our technical requirements by comparing each figure against our figure specifications.

# Author comment: Author response to Decision Letter 3

**DOI:** 10.1371/journal.pone.0352876.r006

15 Jun 2026

Responses to Academic Editor

The academic editor did not raise any specific point

Responses to reviewer

Point 1: Avoid starting sentence with also “145 the unity effect [11]. Also,…”

Our response: we have rephrased the sentence as “Chainey and Humphreys [28] have also shown that…”

Point 2: Avoid personal pronouns “383 Finally, we separated the errors”

Our response: Though the PLOS writing center does not specify a preference for first-person pronouns or the use of active/ passive writing voices, the use of first-person pronouns is permitted and encouraged in both APA and AMA writing styles to improve writing clarity.

Point 3: “474 A planned Bayesian independent samples t-test confirmed that the heart rate of athletes (M = 475 73.660 bpm) was lower than the heart rate of nonathletes (M = 87.389 bpm), BF10 = 2631.” Too many decimal places.

Our response: We have updated our reporting to reflect Lang and Altman’s (2015) SIMPL guidelines (which PLOS references for statistical reporting guidelines) who indicate to round to an "appropriate degree of precision." For bpm (measured with a pulse oximeter that provides whole numbers) this was reduced to one decimal place. Mean age was reported as years and months on all values, so two decimal points is appropriate in this case. Similar logic was maintained for error rate reporting (rounded to 2 decimal places). Reporting BF values is specified in the Open Science Framework as being reported to 2 decimal places for greater than 1, so all BF values were updated as such.

Point 4: Missing space “477 RTfor correct”

Our response: Thank you for pointing out this typo, it has been corrected.

Point 5:

1. Colavita FB. Human sensory dominance. Perception & Psychophysics. 1974 Mar;16(2):409–12. 644 doi:10.3758/BF03203962

645 2. Koppen C, Spence C. Seeing the light: Exploring the Colavita visual dominance effect. Exp Brain Res. 646 2007 Feb 27;180:737-54. doi:10.1007/s00221-007-0894-3

No need to include month of publication in references

Our response: Months were removed from each reference.

Point 6: Probably error on reference number formatting “Colavita FB. Visual dominance and attention in space. Bulletin of the 651 Psychonomic Society. 1982

652 May;19(5):261-2. doi:10.3758/BF03330251 Sinnett S, Soto-Faraco S, Spence C. The co-occurrence of

653 multisensory competition and facilitation. Acta Psychologica. 2008 May;128(1):153–61.

654 doi:10.1016/j.actpsy.2007.12.002”

Our response: Thank you for pointing this duplication, it has been corrected.

Point 7: References often incocnsistent in whether a space after :?

Our response: corrections were made to all relevant articles.

Attachment Submitted filename: Responses_to_reviewer.docx

# Editor report: Decision Letter 3

**DOI:** 10.1371/journal.pone.0352876.r007

**Authors:** Elisa Scerrati

16 Jun 2026

Semantic congruence impacts audiovisual processing in the Colavita effect

PONE-D-25-52734R3

Dear Dr. Desmarais,

We’re pleased to inform you that your manuscript has been judged scientifically suitable for publication and will be formally accepted for publication once it meets all outstanding technical requirements.

Within one week, you’ll receive an e-mail detailing the required amendments. When these have been addressed, you’ll receive a formal acceptance letter and your manuscript will be scheduled for publication.

An invoice will be generated when your article is formally accepted. Please note, if your institution has a publishing partnership with PLOS and your article meets the relevant criteria, all or part of your publication costs will be covered. Please make sure your user information is up-to-date by logging into Editorial Manager at Editorial Manager® and clicking the ‘Update My Information' link at the top of the page. For questions related to billing, please contact billing support.

If your institution or institutions have a press office, please notify them about your upcoming paper to help maximize its impact. If they’ll be preparing press materials, please inform our press team as soon as possible -- no later than 48 hours after receiving the formal acceptance. Your manuscript will remain under strict press embargo until 2 pm Eastern Time on the date of publication. For more information, please contact onepress@plos.org.

Kind regards,

Elisa Scerrati

Academic Editor

PLOS One

Additional Editor Comments (optional):

Reviewers' comments:

# Editor report: Acceptance letter

**DOI:** 10.1371/journal.pone.0352876.r008

**Authors:** Elisa Scerrati

PONE-D-25-52734R3

PLOS One

Dear Dr. Desmarais,

I'm pleased to inform you that your manuscript has been deemed suitable for publication in PLOS One. Congratulations! Your manuscript is now being handed over to our production team.

At this stage, our production department will prepare your paper for publication. This includes ensuring the following:

* All references, tables, and figures are properly cited

* All relevant supporting information is included in the manuscript submission,

* There are no issues that prevent the paper from being properly typeset

You will receive further instructions from the production team, including instructions on how to review your proof when it is ready. Please keep in mind that we are working through a large volume of accepted articles, so please give us a few days to review your paper and let you know the next and final steps.

Lastly, if your institution or institutions have a press office, please let them know about your upcoming paper now to help maximize its impact. If they'll be preparing press materials, please inform our press team within the next 48 hours. Your manuscript will remain under strict press embargo until 2 pm Eastern Time on the date of publication. For more information, please contact onepress@plos.org.

You will receive an invoice from PLOS for your publication fee after your manuscript has reached the completed accept phase. If you receive an email requesting payment before acceptance or for any other service, this may be a phishing scheme. Learn how to identify phishing emails and protect your accounts at https://explore.plos.org/phishing.

If we can help with anything else, please email us at customercare@plos.org.

Thank you for submitting your work to PLOS ONE and supporting open access.

Kind regards,

PLOS ONE Editorial Office Staff

on behalf of

Dr. Elisa Scerrati

Academic Editor

PLOS One
